# Pivotal role for the ESCRT-II complex subunit EAP30/SNF8 in IRF3-dependent innate antiviral defense

**DOI:** 10.1371/journal.ppat.1006713

**Published:** 2017-10-30

**Authors:** Kattareeya Kumthip, Darong Yang, Nan L. Li, Yunzhi Zhang, Meiyun Fan, Aarti Sethuraman, Kui Li

**Affiliations:** 1 Department of Microbiology, Immunology and Biochemistry, University of Tennessee Health Science Center, Memphis, Tennessee, United States of America; 2 Department of Microbiology, Faculty of Medicine, Chiang Mai University, Chiang Mai, Thailand; 3 Institute of Pathogen Biology and Immunology of College of Biology, State Key Laboratory of Chemo/Biosensing and Chemometrics, Hunan University, Changsha, Hunan, China; 4 Department of Infectious Diseases, the Second Affiliated Hospital of Chongqing Medical University, Chongqing, China; 5 Department of Pathology and Laboratory Medicine, University of Tennessee Health Science Center, Memphis, Tennessee, United States of America; Nationwide Children's Hospital, UNITED STATES

## Abstract

The activation of interferon (IFN)-regulatory factor-3 (IRF3), characterized by phosphorylation and nuclear translocation of the latent transcription factor, is central to initiating innate antiviral responses. Whereas much has been learned about the upstream pathways and signaling mechanisms leading to IRF3 activation, how activated IRF3 operates in the nucleus to control transcription of IFNs remains obscure. Here we identify EAP30 (a.k.a, SNF8/VPS22), an endosomal sorting complex required for transport (ESCRT)-II subunit, as an essential factor controlling IRF3-dependent antiviral defense. Depletion of EAP30, but not other ESCRT-II subunits, compromised IRF3-dependent induction of type I and III IFNs, IFN-stimulated genes (ISGs) and chemokines by double-stranded RNA or viruses. EAP30, however, was dispensable for the induction of inflammatory mediators of strict NF-κB target. Significantly, knockdown of EAP30 also impaired the establishment of an antiviral state against vesicular stomatitis virus and hepatitis C virus, which are of distinct viral families. Mechanistically, EAP30 was not required for IRF3 activation but rather acted at a downstream step. Specifically, a fraction of EAP30 localized within the nucleus, where it formed a complex with IRF3 and its transcriptional co-activator, CREB-binding protein (CBP), in a virus-inducible manner. These interactions promoted IRF3 binding to target gene promoters such as IFN-β, IFN-λ1 and ISG56. Together, our data describe an unappreciated role for EAP30 in IRF3-dependent innate antiviral response in the nucleus.

## Author summary

The induction of IFN antiviral response is a hallmark of immediate host immune responses to viral infections. In a majority of cell types, eliciting this intrinsic defense mechanism depends on activation of IRF3, characterized by C-terminal phosphorylation, dimerization and subsequent nuclear translocation of the latent transcription factor. While the molecular pathways and signaling events leading to IRF3 activation have been extensively studied, how activated IRF3 operates in the nucleus to trigger antiviral gene expression remains murky. In this study, we reveal that EAP30, an endosomal sorting complex required for transport (ESCRT)-II subunit, is a novel, pivotal factor controlling IRF3-dependent antiviral defense. We demonstrate that a fraction of EAP30 resides in the nucleus, where it forms a complex with IRF3 and its transcriptional co-activator, CBP, in response to viral insults. These interactions are essential for IRF3 binding to its target gene promoters and subsequent transcription of antiviral genes including those encoding type I and III IFNs and IFN-stimulated genes. Our data unveil a previously unappreciated role for EAP30 in facilitating IRF3-mediated antiviral response in the nucleus and raise the possibility that this novel function of EAP30 may be targeted for developing antiviral interventions to combat infectious diseases.

## Introduction

The early induction of interferons (IFNs) and proinflammatory cytokines and chemokines is a hallmark of host innate immune responses to viral infections [1]. This process starts with the detection of viral components, most notably nucleic acids, by immune sentinel molecules termed pattern recognition receptors (PRRs). Of these, the membrane-bound Toll-like receptors (TLRs) and the cytosolic retinoic-acid-inducible gene I (RIG-I)-like receptors (RLRs, RIG-I and MDA5) are the most characterized [2]. During viral infection, TLR3 located in endolysosomal compartments responds to the presence of double-stranded (ds) RNA [3], which is usually produced as viruses replicate their genomes. TLR3 signals through Toll-interleukin-1 receptor domain-containing adaptor inducing IFN-β (TRIF) leading to the activation of the classical IκB kinase (IKK) complex and IKK-related kinases, TANK-binding kinase 1 (TBK1) and IKKε. These kinases further activate and result in the nuclear translocation of nuclear factor-kappa B (NF-κB) and IFN-regulatory factors (IRFs), transcriptional factors that cooperatively initiate production of type I & III IFNs and inflammatory chemokines such as regulated on activation normal T cell expressed and secreted (RANTES), IFN-γ-inducible protein 10 (IP-10), etc [4–6]. In the cytoplasm, viral dsRNAs or RNAs bearing 5’-triphosphates /diphosphates are detected by the RLRs, which signal through the mitochondrial antiviral signaling protein (MAVS, a.k.a., IPS-1, VISA and Cardif) adaptor. Engagement of the RLR-MAVS pathway also leads to activation of IKK and IKK-related kinases and subsequently, NF-κB and IRFs activation [7–9]. IFNs, once induced through the RLR and/or TLR3 pathways, act in an autocrine/paracrine fashion to upregulate the expression of hundreds of IFN-stimulated genes (ISGs) that collectively establish an antiviral state, halting viral replication and spread [1].

Within the IRF family of transcription factors, IRF3 is crucial for the initial induction of type I IFN response in a majority of cell types [10]. This protein is constitutively expressed as a latent form that predominantly resides in the cytoplasm. Following virus infection, engagement of the RLR or TLR3 pathway culminates in the phosphorylation of specific serine residues in the C-terminal part of IRF3 by TBK1 or IKKε [4, 6]. This post-translational modification relieves IRF3 from its auto-inhibitory conformation, enabling the transcription factor to dimerize and subsequently translocate to the nucleus. Therein, activated IRF3 associates with its transcriptional coactivators, CREB-binding protein (CBP/p300), assembling an “enhanceosome” that stimulates transcription from the IFN-β promoter [4, 11–13]. In addition, the promoters of type III IFNs [14, 15] and a subset of ISGs, e.g., ISG56, ISG15, ZAP and OASL [16, 17], and those of the chemokines RANTES and IP-10 [18, 19], are also transcriptionally controlled by IRF3. In contrast to the rich information available concerning the signaling pathways and mechanisms leading to IRF3 phosphorylation in the cytoplasm, current knowledge on how activated IRF3 operates in the nucleus to induce antiviral gene expression falls short.

The endosomal sorting complex required for transport (ESCRT) machinery participates in numerous cellular processes including multi-vesicular body biogenesis, cellular division, and viral budding, etc. The ESCRT machinery comprises a pathway of five distinct complexes, ESCRTs -0, -I, -II, -III and Vps4, with each containing multiple subunit proteins [20]. Recently, specific ESCRT proteins/complexes have been implicated in the life cycle of several enveloped viruses, including human immunodeficiency type 1 (HIV-1), hepatitis B virus (HBV), and hepatitis C virus (HCV), primarily in viral envelopment/budding process [21–26]. Of particular interest, the ESCRT-II complex, a Y-shaped heterotetramer consisting of two copies of EAP20 (ELL-associated protein of 20 kDa, a.k.a., VPS25) and one subunit each of EAP30 (a.k.a., Vps22 or SNF8) and EAP45 (a.k.a., Vps36) [23, 27], plays an essential part in HBV RNA trafficking and genome encapsidation [25]. The EAP30 subunit also has been reported to regulate HIV-1 genomic RNA trafficking in the cytoplasm, viral gene expression and production [22]. Together, these recent data highlighted a role for ESCRT-II in viral RNA trafficking in the cytoplasm, prompting us to determine whether this complex or its component(s) is involved in innate immune sensing of viruses, which has not been investigated previously. As described below, our study uncovered an unappreciated role of EAP30 in IRF3-dependent antiviral responses. Surprisingly, we found that EAP30 acts in the nucleus to execute this novel function, by facilitating the binding of activated IRF3 to CBP and its target gene promoters.

## Results

### EAP30 is required for induction of innate immune genes following engagement of TLR3 and RIG-I antiviral pathways

**Fig 1:**
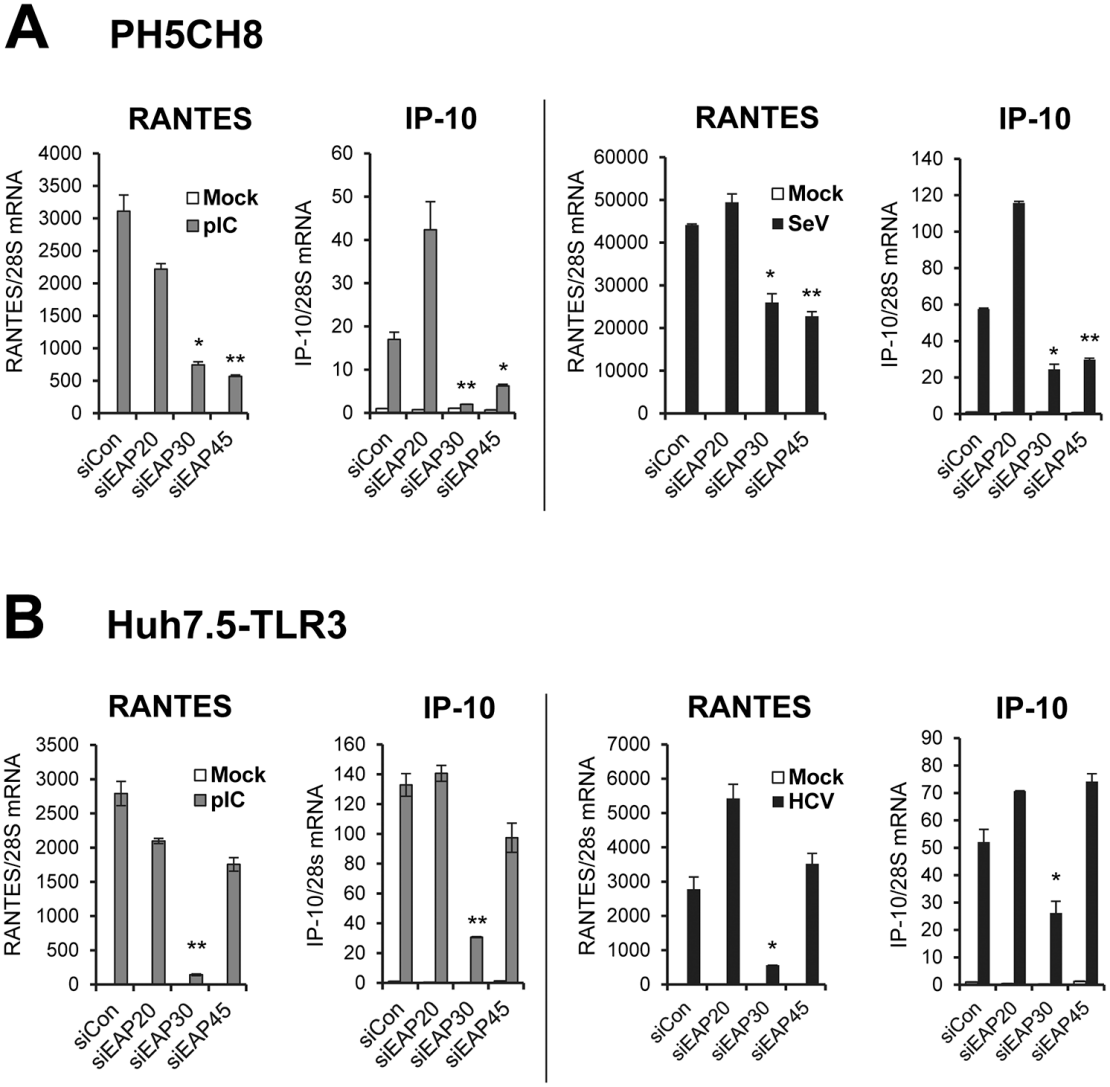
Impact of ESCRT-II knockdown on dsRNA- or virus-induced chemokine expression. **(A)** qPCR analysis of RANTES and IP-10 mRNA levels in PH5CH8 cells transfected with indicated siRNAs and mock-treated (empty bars), stimulated by 10 μg/ml of poly(I:C) for 6 h (grey bars), or infected with SeV at 160 HAU/ml for 8 h (black bars). **(B)** qPCR analysis of RANTES and IP-10 mRNA levels in Huh7.5-TLR3 cells transfected with indicated siRNAs and mock-treated (empty bars), stimulated by 10 μg/ml of poly(I:C) for 6 h (grey bars), or infected with HCV-JFH1 (MOI = 0.1) for 56 h (black bars). For the HCV groups cells were infected for 8 h prior to siRNA transfection for additional 48 h. “*” and “**” denote statistical differences exist as compared with control siRNA-transfected cells with a *P*-value of < 0.05 and < 0.01, respectively.

To determine whether the ESCRT-II complex proteins are involved in innate immune response to viral infections, we studied the impact of depleting each subunit, i.e., EAP20, EAP30 and EAP45, on induction of innate immune genes following stimulation by poly(I:C) or infection with Sendai virus (SeV), well-characterized inducers for TLR3 and RIG-I signaling pathways, respectively [4, 9, 28]. We first used PH5CH8 cells, which were derived from non-neoplastic hepatocytes transformed with SV40 large T antigen. This cell line harbors intact TLR3 and RIG-I/MDA5 antiviral signaling pathways similar to primary human hepatocytes [28, 29]. We transfected the cells with a synthetic siRNA duplex specifically targeting each human ESCRT-II subunit or a non-targeting, negative control siRNA, then stimulated the cells by poly(I:C) or SeV prior to quantitative reverse-transcription PCR (qPCR) analysis of RANTES and IP-10 mRNA expression. Both chemokines are known to be highly induced early after viral infections, and their transcription is under coordinate control of IRF3 and NF-kB [18, 19, 29]. qPCR data demonstrated that the ESCRT-II siRNAs were highly effective, curtailing expression of their cognate targets by ~90%, with or without poly(I:C) or SeV stimulation (S1A Fig). We note that expression of all three ESCRT-II subunits was unaltered by poly(I:C) or SeV stimulation. Immunoblot analyses showed that these siRNAs also efficiently inhibited the expression of their cognate target proteins (S1B Fig). Compared to cells receiving control siRNA, cells transfected with EAP30 or EAP45 siRNA were significantly impaired for poly(I:C)- or SeV-induced RANTES and IP-10 mRNA expression (Fig 1A). No such effect, however, was observed in EAP20 siRNA-transfected cells. We confirmed by ELISA that SeV-induced RANTES production in culture supernatants was significantly lower in EAP30 knockdown cells than in cells transfected with control siRNA (S2 Fig). We examined SeV RNA levels in siRNA-transfected cells and demonstrated impaired induction of chemokines in EAP30- or EAP45-depleted cells was not due to reduced viral replication (S3 Fig).

To determine whether the observation holds true in a different virus infection setting and in other cell types, we investigated the effects of ESCRT-II knockdown on chemokines production in HCV-infected Huh7.5-TLR3 cells. These hepatoma cells are stably reconstituted for TLR3 expression and signaling but are defective for RIG-I [30]; they respond to HCV infection by upregulating RANTES and IP-10 expression through the TLR3 pathway [29]. Efficient, siRNA-mediated knockdown of EAP20, EAP30, and EAP45 in Huh7.5-TLR3 cells was confirmed by qPCR (S4 Fig). As shown in Fig 1B, poly(I:C)- or HCV-induced transcription of RANTES and IP-10 mRNAs was significantly decreased in EAP30 knockdown cells. In contrast, silencing EAP20 or EAP45 did not exhibit an inhibitory effect. HCV RNA levels in cells transfected with control, EAP20 or EAP45 siRNAs were comparable, while significantly higher in EAP30 knockdown cells (S5 Fig), suggesting that the decrease in chemokine induction following EAP30 knockdown was not attributed to reduced viral replication or pathogen-associated molecular pattern (PAMP) expression.

Taken together, these data suggest that EAP30 is implicated in innate immune responses to viral infections triggered through either TLR3 or RIG-I while EAP45 contributes particularly to signaling through the RIG-I pathway.

### EAP30 is essential for activation of the IFN-β promoter and acts downstream of IRF3

**Fig 2:**
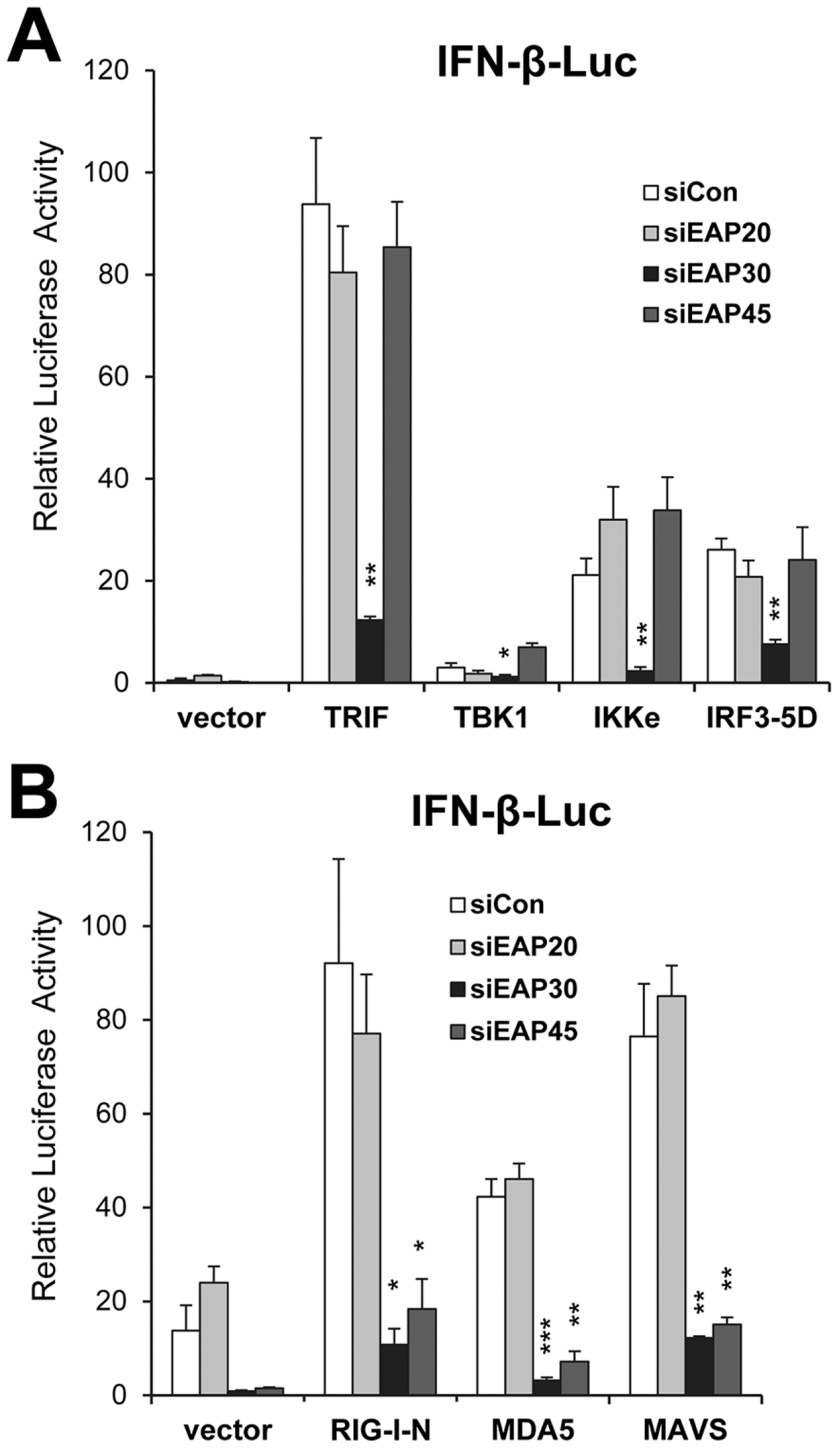
Impact of ESCRT-II knockdown on activation of the IFN-β promoter by ectopic expression of various signaling proteins in TLR3 and RIG-I pathways. **(A)** IFN-β promoter activity following ectopic expression of control vector, TRIF, TBK1, IKKε or IRF3-5D in PH5CH8 cells that had been transfected with the indicated siRNAs. **(B)** IFN-β promoter activity following ectopic expression of control vector, RIG-I CARD (RIG-I-N), MDA5, or MAVS in PH5CH8 cells that had been transfected with the indicated siRNAs.“*”, “**”, and “***” denote statistical differences exist as compared with control siRNA-transfected cells with a *P*-value of < 0.05, < 0.01 and <0.001, respectively.

Apart from inflammatory cytokine/chemokine production, a salient feature of the innate antiviral responses is the early induction of type I IFNs. We determined how depletion of each ESCRT-II subunit influenced activation of the IFN-β promoter via the TLR3 and RIG-I/MDA5 pathways (illustrated in S6A Fig). Following overexpression of individual signaling proteins downstream of the TLR3 pathway including TRIF, TBK1, IKKε, and IRF3-5D (a constitutively active, phospho-mimetic IRF3 mutant), upregulation of the IFN-β promoter was clearly observed in control siRNA transfected cells, as was in cells transfected with EAP20 or EAP45 siRNA. In contrast, knockdown of EAP30 strongly inhibited IFN-β promoter activation following ectopic expression of any of the 4 signaling molecules (Fig 2A). When we examined activation of the IFN-β promoter via the RIG-I/MDA5 pathway by overexpressing RIG-I, MDA5, or the downstream adaptor, MAVS, we observed strong inhibitory effects after we silenced expression of EAP30 or EAP45, but not that of EAP20, as compared with control siRNA (Fig 2B). Immunoblot analyses confirmed efficient expression of MAVS and two downstream signaling components (IKKε and IRF3-5D) that are shared by TLR3 and RLR pathways and proximal to IFN-β induction in cells receiving EAP30 or EAP45 siRNA, as compared with control- or EAP20 siRNA-transfected cells (S6B and S6C Fig). Collectively, these results indicate that EAP30 is an essential contributor to TLR3 and RIG-I/MDA5 signaling pathways leading to IFN-β expression and acts downstream of the IRF3 kinases and the IRF3 phosphorylation step, whereas EAP45 is specifically involved in IFN-β activation via the RIG-I/MDA5-MAVS pathway. These data also are in line with the chemokine production results following dsRNA and viral stimuli (Fig 1). In subsequent investigations, we focused on dissecting the role of EAP30.

### EAP30 plays a critical part in IRF3-dependent innate immune response but has no role in viral activation of NF-kB-dependent genes

**Fig 3:**
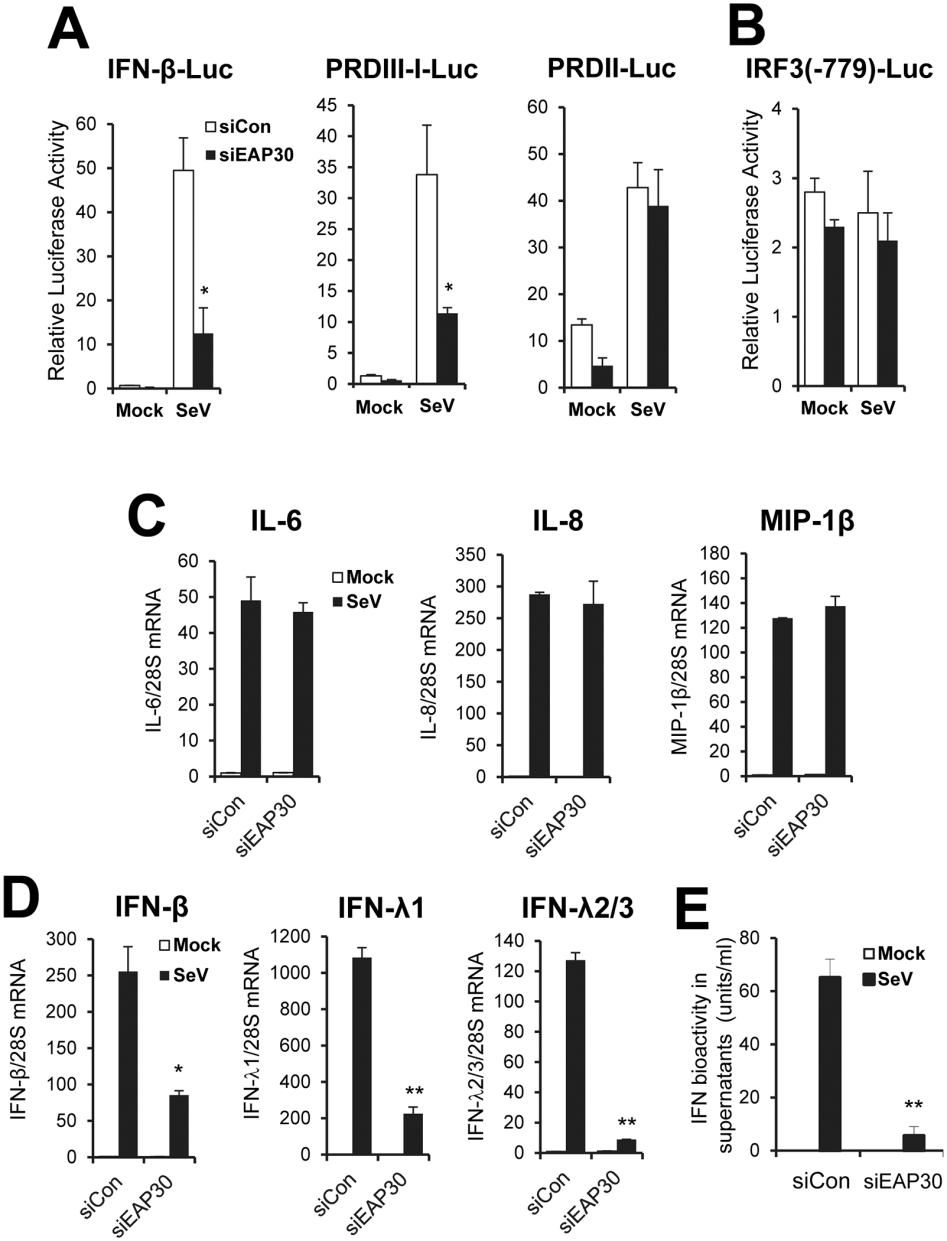
The impairment in viral induction of type I and type III IFNs following EAP30 knockdown is attributed to an IRF3-dependent and NF-κB-independent mechanism. **(A)** Activation of the IFN-β promoter, IRF3-dependent PRDIII-I element, and NF-κB-dependent PRDII element by SeV in PH5CH8 cells transfected with control (empty bars) or EAP30 (solid bars) siRNA. **(B)** Activity of the human IRF3 promoter (IRF3(-779)) in PH5CH8 cells transfected with control siRNA (empty bars) or EAP30 siRNA (solid bars), and mock-infected or infected with SeV. **(C)** qPCR analysis of IL-6, IL-8, and MIP-1β mRNA levels in PH5CH8 cells transfected with control siRNA or EAP30 siRNA, and mock-infected (empty bars)or infected with SeV (solid bars). **(D)** qPCR analysis of IFN-β, IFN-λ1, and IFN-λ2/3 expression in control siRNA- or EAP30 siRNA-transfected PH5CH8 cells, mock-infected (empty bars)or infected with SeV (solid bars). **(E)** Production of IFN antiviral activity in culture supernatants of PH5CH8 cells under experimental conditions of **(D)**. “*” and “**” denote statistical differences exist as compared with control siRNA-transfected cells with a *P*-value of < 0.05 and < 0.01, respectively.

**Fig 4:**
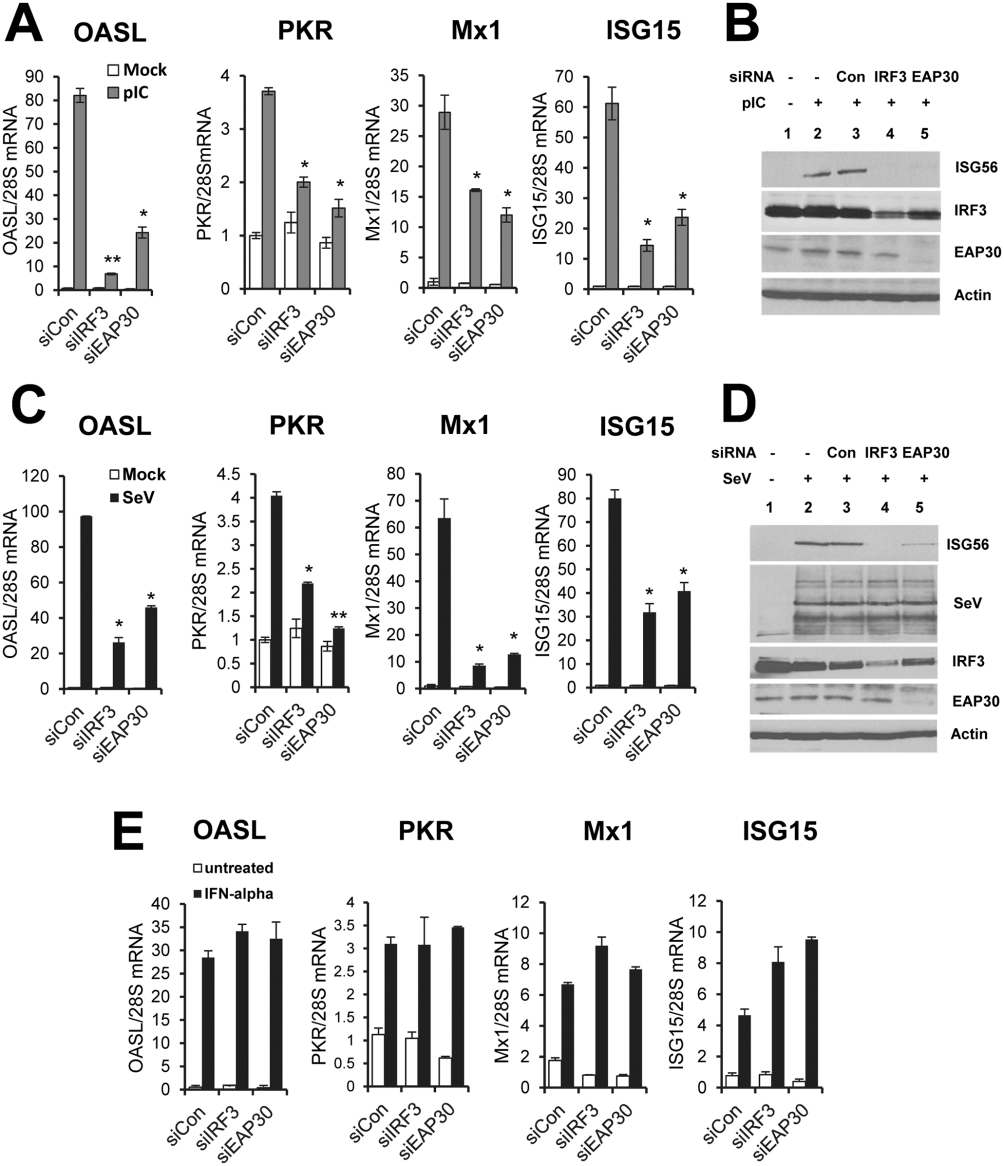
EAP30 silencing decreases poly(I:C)- and SeV-induced ISGs expression but not ISG induction by IFN-α. **(A)** qPCR analysis of OASL, PKR, MX1 and ISG15 mRNA levels in PH5CH8 cells transfected with control, IRF3, or EAP30 siRNA for 48 h, and mock-treated (empty bars)or stimulated with poly(I:C) (10 μg/ml) for additional 8 h (gray bars). **(B)** Immunoblotting of ISG56, IRF3, EAP30 and actin proteins under experimental conditions of (A). **(C)** qPCR quantification of OASL, PKR, MX1 and ISG15 mRNA levels in PH5CH8 cells transfected with control, IRF3, or EAP30 siRNA for 48 h, and mock-treated (empty bars)or infected with SeV (160 HAU/ml) for additional 8 h (solid bars). **(D)** Immunoblotting of ISG56, SeV, IRF3, EAP30 and actin proteins under experimental conditions of (C). **(E)** qPCR quantification of OASL, PKR, MX1 and ISG15 mRNA levels in PH5CH8 cells transfected with control, IRF3, or EAP30 siRNA for 48 h, and mock-treated (empty bars)or stimulated with IFN-α (100 U/ml) for additional 8 h (solid bars). “*” and “**” denote statistical differences exist as compared with control siRNA-transfected cells with a *P*-value of < 0.05 and < 0.01, respectively.

To determine the mechanism by which EAP30 contributes to the IFN response, we performed luciferase reporter assay examining the effects of EAP30 knockdown on viral activation of the IFN-β promoter and the IRF3-responsive PRDIII-I and NF-κB-dependent PRDII elements within the promoter. Interestingly, depletion of EAP30 significantly reduced SeV-induced activation of the IFN-β promoter and the IRF3-dependent PRDIII-I element, but left activation of the NF-κB-dependent PRDII motif unaffected (Fig 3A). This result is consistent with our earlier observation that EAP30 acts downstream of IRF3 phosphorylation (Fig 2A). The specific inhibition on IRF3-dependent promoter was not due to a decrease in IRF3 expression, as EAP30 depletion had no effect on the native IRF3 promoter (Fig 3B), which is constitutively active and whose activity does not change after viral infections [31]. Moreover, the levels of endogenous IRF3 transcript (S7 Fig) and protein (see below, Fig 4B and 4D) were not altered by EAP30 knockdown. To further confirm that EAP30 is dispensable for viral activation of NF-κB-dependent gene expression, we quantified transcript for IL-6, IL-8, and MIP-1β. SeV strongly upregulated the expression of all 3 strict NF-kB target genes, without a discernable difference between control and EAP30 siRNA-transfected cells (Fig 3C). Examining the expression of 7 additional genes (TRAF1, IL32, RELB, P100, BIRC3, COX2 and SGPP2) whose expression is predominantly controlled by NF-kB [32–36] led to the same conclusion (S8 Fig). Under the same experimental conditions, EAP30 knockdown led to profound reduction in virus-induced IFN-β mRNA abundance (Fig 3D). In addition, the upregulation of IFN-λ1 (a.k.a.,IL29) and IFN-λ2/3 (a.k.a., IL28A/B) mRNAs, which is also critically dependent on IRF3, was impaired by 80% and 93%, respectively, following EAP30 silencing (Fig 3D). Quantification of IFN antiviral activity in culture supernatants by a bioassay based on VSV plaque reduction revealed that SeV-induced IFN production was diminished in EAP30 knockdown cells, in comparison to cells transfected with control siRNA (Fig 3E).

Q-PCR analysis showed that knockdown of EAP30 resembled the effect of IRF3 silencing in that it substantially curtailed the induction of 4 well-characterized ISGs, OASL, PKR, MX1, and ISG15, by poly(I:C) (Fig 4A) or SeV (Fig 4C). The same could be said regarding the induction of ISG56 protein (Fig 4B and 4D). Immunoblotting data also demonstrated that EAP30 siRNA did not affect IRF3 protein expression, as compared to control siRNA (Fig 4B and 4D, compare lane 5 vs lane 3). Moreover, depletion of EAP30 significantly impaired SeV-induced upregulation of five additional ISGs, i.e., OAS1, ISG20, RSAD2 (a.k.a., viperin), IRF7 and IRF1 (S9 Fig), further ruling out a gene-specific effect of EAP30 on ISG induction. Altogether, these results suggest that EAP30 is specifically required for IRF3-dependent innate immune response, including activation of type I and III IFN production and induction of ISGs.

To corroborate these findings in a different cell type, we developed HEK293 cells with stable knockdown of EAP30 (referred to as 293-shEAP30 cells) by lentiviral-mediated transfer of a shRNA construct specifically targeting EAP30. Cells transduced with a non-targeting, scrambled control shRNA (referred to as 293-shCon cells) served as a negative control. As shown in S10 Fig, there was significant reduction in EAP30 protein abundance in 293-shEAP30 cells as compared with 293-shCon cells. This was correlated with diminished viral induction of ISG15, IFIT3 and MDA5 proteins. Viral upregulation of these 3 ISGs is controlled by IRF3 [16, 37], similar to that of ISG56. In stark contrast, the upregulation of P100 and TRAF1 proteins by SeV infection was comparable between 293-shEAP30 and 293-shCon cells (S10 Fig), again suggesting that EAP30 is dispensable for viral activation of the NF-κB arm of host responses. Of note, we did not observe any difference in cell viability or proliferation rate between 293-shCon cells and 293-shEAP30 cells (S11 Fig). Thus, the impaired ISG induction following EAP30 knockdown cannot be attributed to a general effect on cellular physiology.

### EAP30 is dispensable for IFN signaling through the Jak-Stat pathway

Although a small subset of ISGs such as ISG56, ISG15, and OASL, etc, can be transcriptionally upregulated by activated IRF3 directly, majority of the ~300 known ISGs is regulated by the paracrine/autocrine action of IFNs through the IFN receptors. Thus, we next investigated whether EAP30 has a role in IFN signaling through the Jak-Stat pathway. To this end we examined ISG induction following IFN-α stimulation (Fig 4E). In control siRNA transfected cells, IFN-α upregulated transcription of OASL, PKR, MX1, and ISG15 to various degrees. This effect was not inhibited by EAP30 or IRF3 siRNA. Likewise, we found IFN-α was similarly effective in upregulating the protein levels of ISG15 and IFIT3 between 293-shCon and 293-shEAP30 cells (S12A Fig). Consistent with the ISG protein data, pretreatment of IFN-α acted comparably in 293-shCon and 293-shEAP30 cells in protecting cells from subsequent challenge by VSV-Luc (S12B Fig), a recombinant vesicular stomatitis virus (VSV) encoding luciferase that serves as a readout of viral replication. In aggregate, we conclude that EAP30’s contribution to innate immune responses is prior to the induction of IFNs and that EAP30 is not involved in downstream IFN signaling, once IFNs are produced.

### EAP30 is required for establishment of an antiviral state

**Fig 5:**
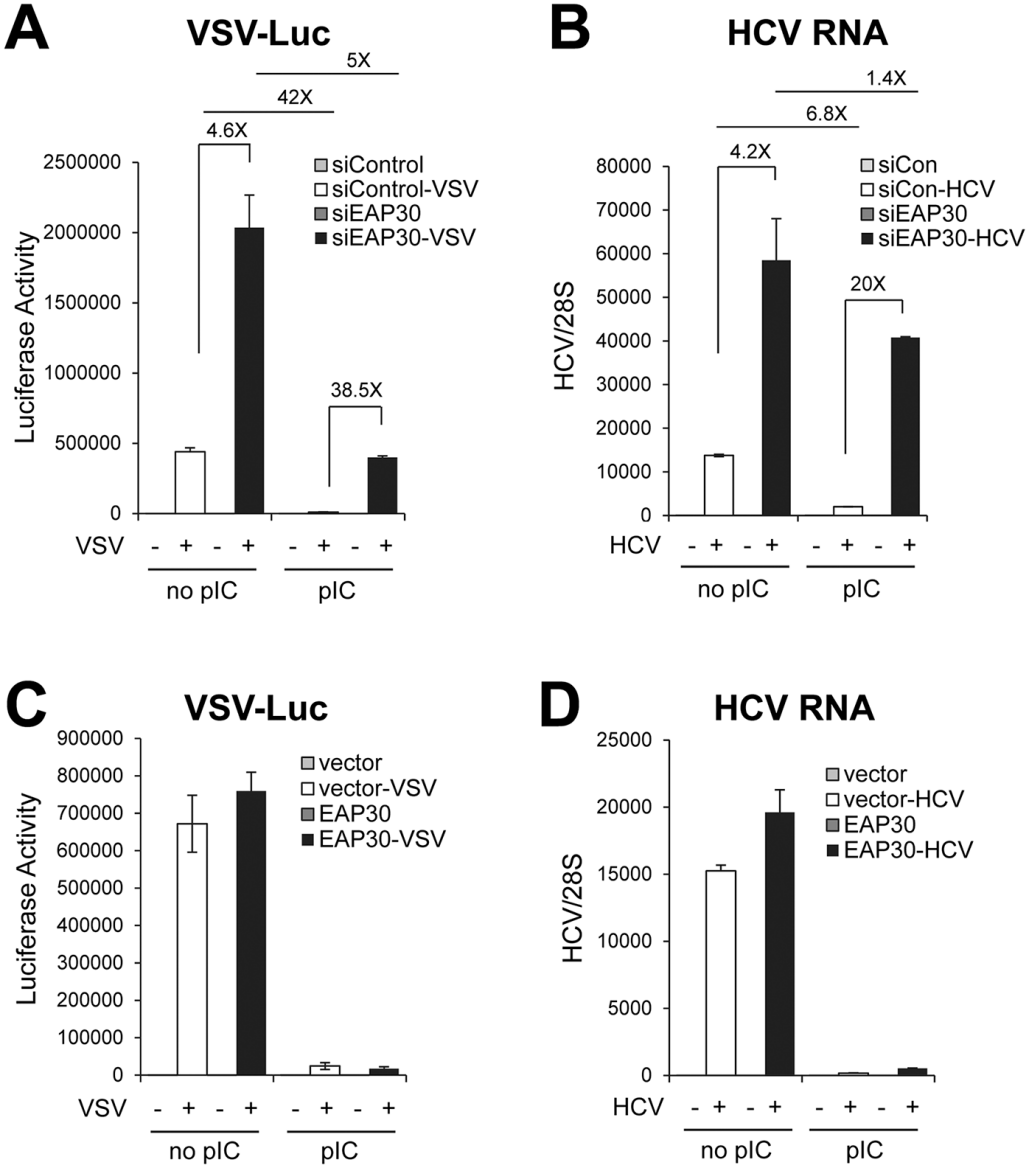
Knockdown of EAP30 compromises cellular innate antiviral defense. **(A)** PH5CH8 cells transfected with control or EAP30 siRNA for 48 h were incubated with or without poly(I:C) (20 μg/ml) for 16 h prior to challenge with VSV-Luc (MOI = 0.1) for 6 h. Luciferase assay was performed to monitor VSV replication levels. **(B)** Control siRNA- or EAP30 siRNA-transfected-Huh7-TLR3 cells were mock-treated or transfected with 2 μg of poly(I:C) for 16 h prior to HCV-JFH1 infection (MOI = 0.05) for 48 h. Intracellular HCV RNA levels (relative to 28S rRNA) were quantified by qPCR. **(C)** PH5CH8 cells transiently overexpressing control vector or EAP30 were incubated with poly(I:C) for 16 h prior to challenge with VSV-Luc (MOI = 0.1) for 6 h. Luciferase assay was performed to monitor VSV replication levels. **(D)** Control vector- or EAP30-overexpressing Huh7-TLR3 cells were mock-treated or transfected with poly(I:C) for 16 h prior to HCV-JFH1 infection (MOI = 0.05) for 48 h. Intracellular HCV RNA levels (relative to 28S rRNA) were quantified by qPCR.

Having shown that EAP30 is specifically required for IRF3-dependent innate antiviral gene expression, we sought to directly assess the biological function of this protein in antiviral defense. We transfected PH5CH8 cells with control or EAP30 siRNA, then mock-treated or stimulated cells with poly(I:C), followed by infecting them with VSV-Luc. In the absence of poly(I:C) pretreatment, VSV-Luc replicated robustly in the cells as indicated by high levels of luciferase activity. Compared to control siRNA transfected cells, cells with EAP30 knockdown supported 4.6-fold higher level of VSV replication (Fig 5A, compare filled bar with empty bar in the “no pIC” group). Prior poly(I:C) stimulation induced an antiviral state, reducing VSV-Luc replication by 42-fold in control siRNA-transfected cells (compare the two empty bars between “pIC” and “no pIC” groups). EAP30 knockdown severely compromised poly(I:C)-induced antiviral state, enabling 38.5-fold higher viral replication than in control siRNA-transfected, poly(I:C)-stimulated cells (compare filled bar with empty bar in the “pIC” group). Of note, the protective effect of poly(I:C), as gauged by the fold-reduction in VSV-encoded luciferase activity, was attenuated by ~88% in EAP30 knockdown cells (~5-fold, compare the two filled bars between “pIC” and “no pIC” groups) compared with that in control siRNA-transfected cells (42-fold). These data provide direct evidence that endogenous EAP30 is essential for the establishment of an antiviral state in the cell. Confirming that the observation is not a cell-specific or virus-specific phenomenon, we found that knockdown of EAP30 also resulted in significant weakening of antiviral defense against HCV infection in hepatoma cells with and without poly(I:C) pre-treatment, enabling higher levels of intracellular HCV RNA replication than in cells transfected with control siRNA (Fig 5B).

We next investigated whether overexpression of EAP30 enhances cellular antiviral defense. Compared with empty control vector-transfected cells, PH5CH8 cells transiently overexpressing EAP30 supported similar levels of VSV-Luc replication, and poly(I:C)-mediated antiviral effect was also comparable (Fig 5C). Similar results were obtained comparing HCV RNA replication in HCV-infected hepatoma cells ectopically expressing control vector or EAP30 (Fig 5D). We conclude from these experiments that while EAP30 is required for establishment of an antiviral state in the cells, overexpression of EAP30 *per se* is not sufficient for activating or augmenting antiviral defense.

### EAP30 is dispensable for virus-induced IRF3 phosphorylation, dimerization, and nuclear translocation

**Fig 6:**
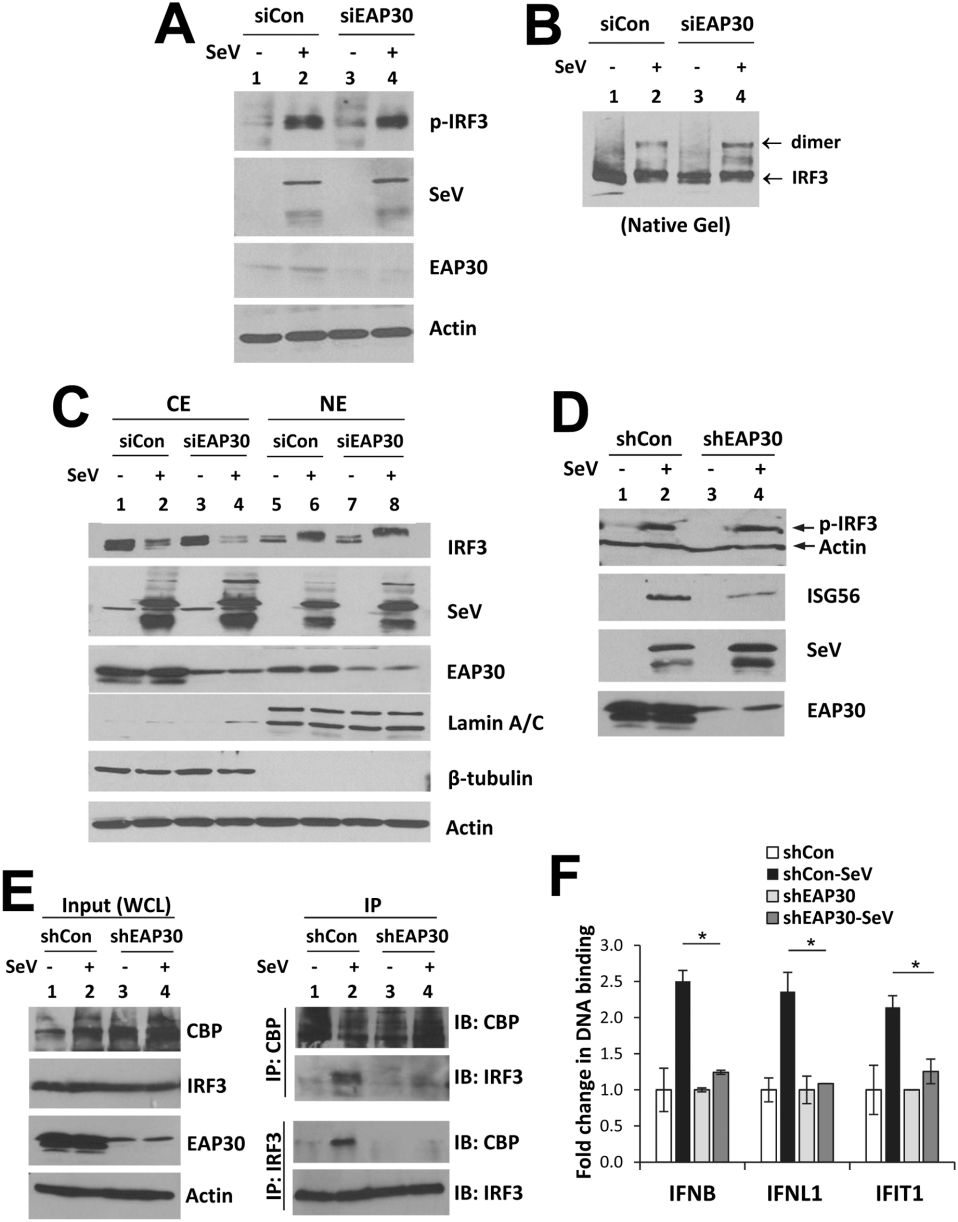
Knockdown of EAP30 does not affect virus-induced IRF3 phosphorylation, dimerization or nuclear translocation, but impairs IRF3-CBP complex formation and IRF3 binding to target gene promoters. **(A)** Immunoblotting of phosphorylated-IRF3 (p-IRF3), SeV, EAP30 and action levels in PH5CH8 cells transfected with control or EAP30 siRNA for 48 h and mock-infected or infected with SeV for additional 8 h. **(B)** Immunoblotting of IRF3 monomer and dimer forms following native PAGE of the samples shown in (A). **(C)** Immunoblotting of IRF3, SeV, EAP30, lamin A/C (nuclear protein marker), β-tubulin (cytoplasmic protein marker), and actin loading control in cytoplasmic (CE) and nuclear (NE) fractions of PH5CH8 cells transfected with control or EAP30 siRNA and mock-infected or infected with SeV. **(D)** Immunoblotting of p-IRF3, actin, ISG56, SeV, and EAP30 in HEK293-shEAP30 and HEK293-shCon cells mock-infected or infected with SeV for 8 h. **(E)** Whole cells lysate (WCL) were collected from HEK293-shEAP30 and HEK293-shCon cells that were mock-infected or infected with SeV for immunoblotting of CBP, IRF3, EAP30 and actin (left panel) and co-IP analysis of virus-induced CBP-IRF3 association (right panel).**(F)** ChIP analysis of IRF3 binding to IFNβ, IFNL1, and IFIT1 (ISG56) promoters in nuclear extracts of HEK293-shEAP30 and HEK293-shCon cells that were mock-infected or infected with SeV. The ChIP-enriched DNA levels were analyzed by qPCR and normalized to input DNA, followed by subtraction of nonspecific binding determined using control IgG. “*” denotes statistical differences exist with a *P*-value of < 0.05.

Although our earlier gene reporter data generated using the phospho-mimetic IRF3-5D (Fig 2A) had suggested that EAP30’s contribution to IFN activation is downstream of IRF3, they did not reveal directly whether EAP30 depletion affects virus-induced activation of IRF3. To clarify, we determined the status of SeV-induced phosphorylation, dimerization and nuclear translocation of IRF3 in cells transfected with control or EAP30 siRNA. Immunoblotting data demonstrated that the abundance of phosphor-IRF3 induced by SeV infection was comparable in PH5CH8 cells with and without EAP30 knockdown (Fig 6A, compare lanes 4 vs 2). Likewise, SeV-induced IRF3 dimerization was not disturbed by EAP30 depletion (Fig 6B, compare lanes 4 vs 2). In addition, cytoplasmic (CE) and nuclear (NE) fraction assay revealed that knockdown of EAP30 did not influence the accumulation of IRF3 in the nucleus of SeV-infected cells (Fig 6C, compare lanes 8 vs 6). Interestingly, a fraction of EAP30 was found to localize in the nucleus, and the nuclear abundance of EAP30 was not different before and after SeV infection (compare lanes 6 vs 5 and 8 vs 7, respectively). This observation was confirmed by confocal fluorescence microscopy, which revealed that in PH5CH8 cells endogenous EAP30 was indeed distributed to both cytoplasm and nucleus, prior to and after SeV infection (S13 Fig). IRF3 immunofluorescence staining data demonstrated that SeV-induced IRF3 nuclear translocation occurred in ~95% and ~98% of PH5CH8 cells with or without EAP30 knockdown (S14 Fig), again suggesting IRF3 nuclear translocation is EAP30-independent. Collectively, these data are in keeping with the IRF3-5D overexpression data (Fig 2A), and lend further support to the notion that EAP30 regulates a step downstream of IRF3 activation, likely after phosphorylated IRF3 has moved into the nucleus.

### EAP30 is required for virus-induced association of IRF3 with its transcriptional co-activator, CBP, and for binding of activated IRF3 to its target promoters

To determine the exact role of EAP30 in IRF3-dependent antiviral response, we conducted comparative studies in293-shEAP30 cells and293-shCon cells. Immunoblot analysis revealed profound loss of EAP30 protein in 293-shEAP30 cells as compared with 293-shCon cells (Fig 6D, compare lanes 3 and 4 vs 1 and 2), concomitant with diminished ISG56 induction by SeV (compare lanes 4 vs 2). This was observed despite that virus-induced IRF3 phosphorylation was intact in EAP30 knockdown cells (compare lanes 4 vs 2). These data thus confirmed in an additional cell line that EAP30 is not required for IRF3 phosphorylation.

In addition to permitting IRF3 dimerization and translocation to the nucleus, virus-induced phosphorylation of IRF3 C-terminal serine residues accommodates binding of the transcription factor to its co-activators, such as CBP, thereby forming an enhanceosome that activates IFN-β promoter transcription [11–13]. To evaluate whether EAP30 is required for IRF3 binding to CBP, we conducted Co-IP experiments in 293-shCon and 293-shEAP30 cells, mock-infected or infected with SeV for 8 h. We found that SeV infection induced a complex formation between endogenous IRF3 and CBP in HEK293-shCon control cells. However, this was diminished in 293-shEAP30 cells (Fig 6E).

To characterize the functional consequence of impaired IRF3-CBP complex formation as a result of EAP30 depletion, we further performed ChIP assay quantifying the IRF3 binding to three well-known IRF3 target gene promoters, IFN-β, IFNL1, and IFIT1 (a.k.a., ISG56). We found that SeV infection triggered significant IRF3 binding to IFN-β, IFNL1, and IFIT1 promoters in 293-shCon cells, but was barely able to do so in 293-shEAP30 cells (Fig 6F). These results imply that EAP30 is required for IRF3 binding to CBP and subsequently its target gene promoters, explaining why EAP30 knockdown was associated with impaired viral induction of type I and III IFNs and ISGs despite intact IRF3 activation.

Since our earlier data (Fig 3 and S8 Fig) had shown that EAP30 knockdown had no demonstrable effect on viral induction of NF-κB-dependent and IRF3-independent genes, we also performed ChIP assay to evaluate SeV-induced NF-κB binding in 293-shEAP30 cells in comparison with 293-shCon cells (S15 Fig). The data showed that the increased p65 binding to NF-κB motifs within IL8, CXCL1 and IL32 promoters following SeV infection was not impaired by EAP30 knockdown.

### EAP30 forms a complex with IRF3 and CBP

**Fig 7:**
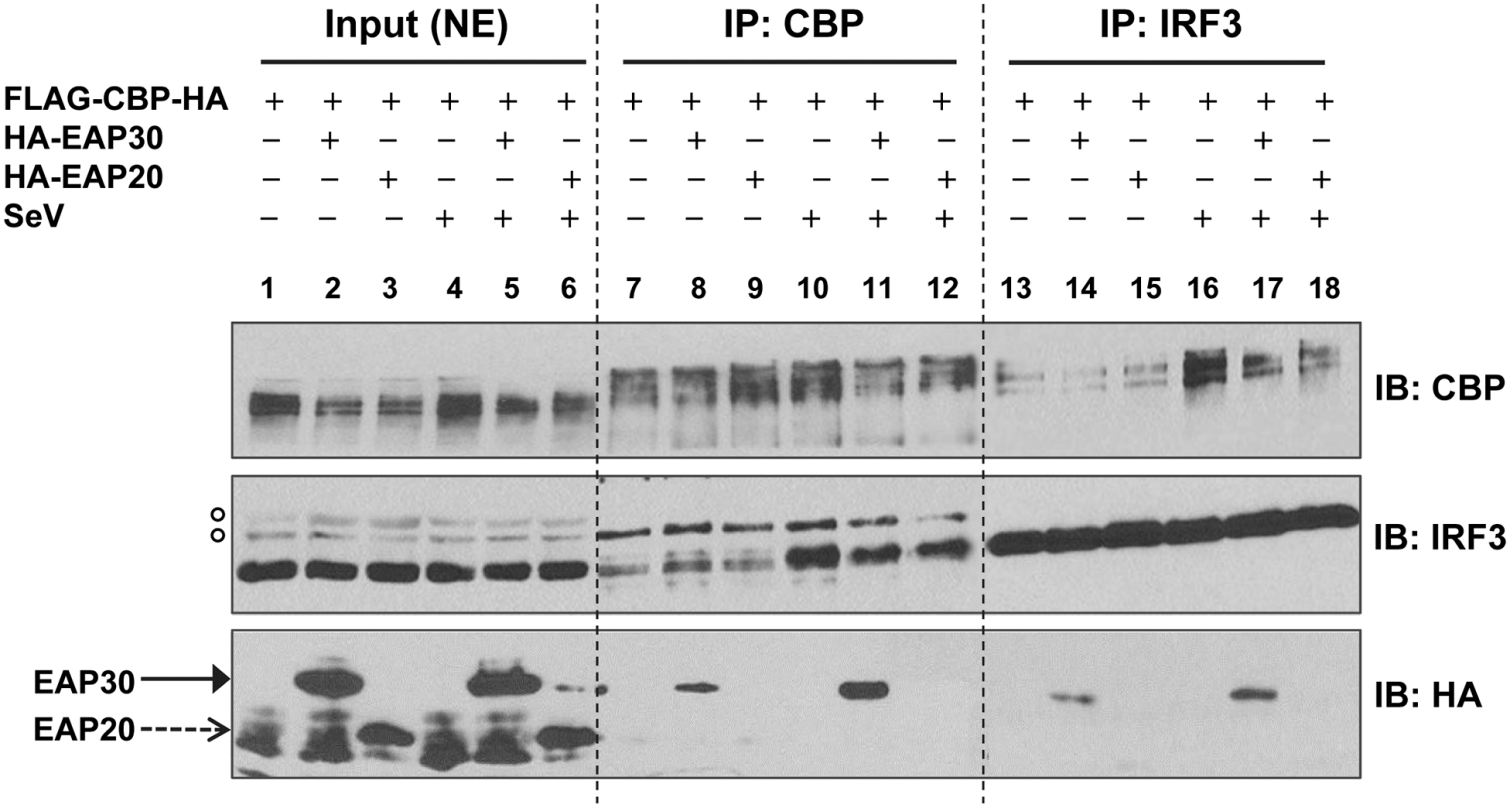
EAP30 forms a complex with IRF3 and CBP. HEK293FT cells were cotransfected with empty vector, HA-EAP30 or HA-EAP20, respectively, along with a FLAG-CBP-HA construct for 48 h, followed by infection with SeV for additional 8 h. Nuclear extracts (NE) were isolated and used for immunoblotting (lanes 1–6) and co-IP analysis (lanes 7–18). Immunoprecipitation was performed by using anti-CBP pAb (lanes 7–12) or anti-IRF3 pAb (lanes 13–18), followed by immunoblotting with anti-CBP, anti-IRF3, and anti-HA antibodies. Empty circles shown on the left to the IRF3 immunoblot denote non-specific bands that served as a loading control.

To determine how EAP30 regulates the IRF3-CBP complex formation and binding of IRF3 to target promoters, we tested the hypothesis that EAP30 acted as an interaction partner facilitating IRF3 binding to CBP and the target promoters in the nucleus. Co-IP experiments were performed using nuclear extracts of HEK293FT cells to evaluate the potential interactions between EAP30 and CBP, EAP30 and IRF3, prior to and after SeV infection (Fig 7). It was found that EAP30 weakly interacted with CBP (lane 8) and IRF3 (lane 14) in uninfected cells, and that both associations were notably stronger after SeV infection (compare lanes 11 vs 8 and lanes 17 vs 14, respectively). In contrast, EAP20 did not form a complex with either CBP (lanes 9 and 12) or IRF3 (lane 15 and 18), regardless of SeV infection status, suggesting that the EAP30 associations with CBP and IRF3 were both specific. Moreover, immunoblotting of CE and NE fractions of the cell lysates demonstrated that indeed EAP30 not only localized to the cytoplasm but also was present in the nuclear fraction, and that the abundance of nuclear EAP30 protein did not obviously change following SeV infection (S16 Fig). These data are in agreement with our earlier CE/NE fractionation data generated from PH5CH8 cells (Fig 6C). Both datasets thus support a role of EAP30 in the nucleus.

### EAP30 synergizes with IRF3 and CBP to induce IFN antiviral response

**Fig 8:**
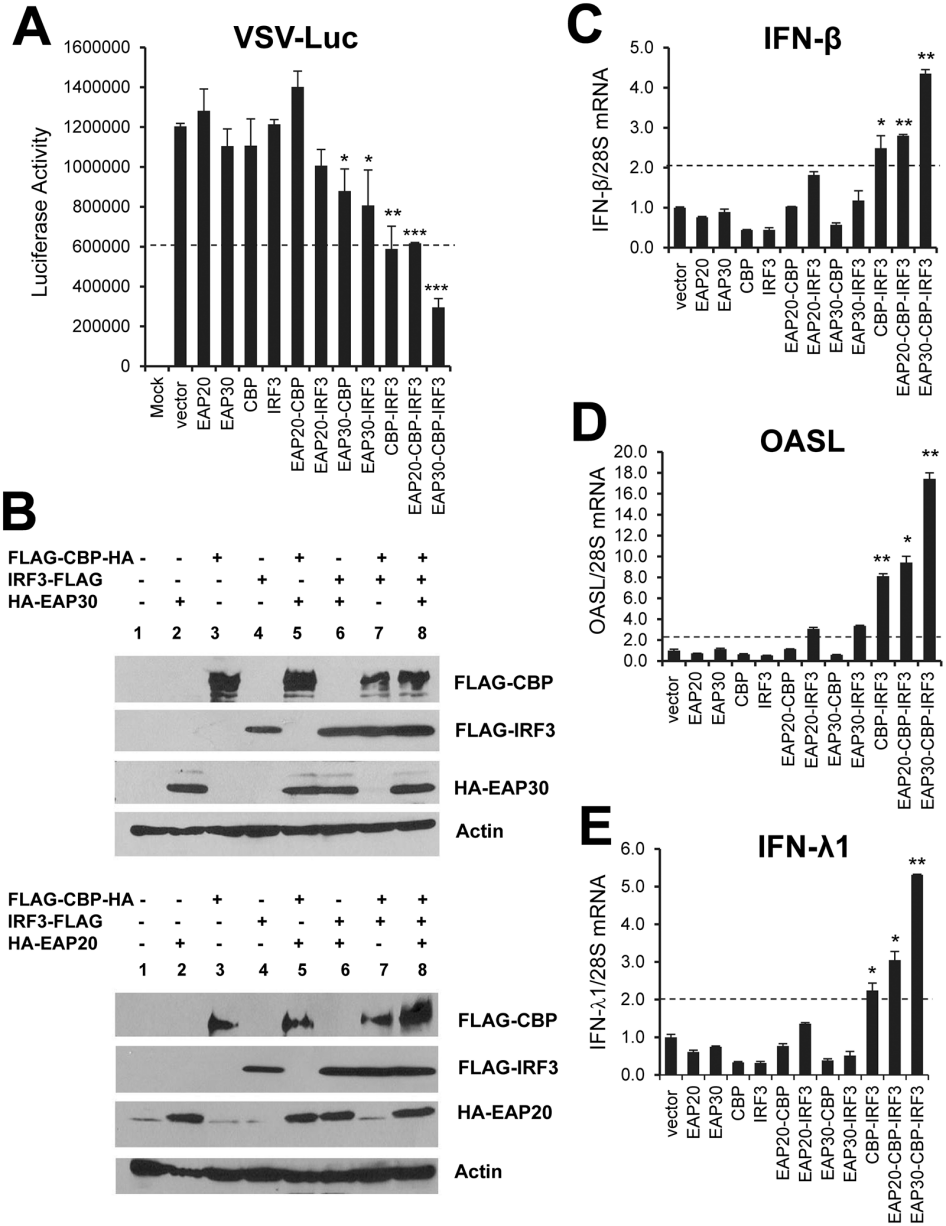
EAP30 synergizes with IRF3 and CBP to induce IRF3-dependent antiviral gene expression, resulting in an enhanced antiviral response. **(A)** HEK293 (5x10^4^) cells were transfected with 100 ng of each plasmid or various double/triple plasmid combinations as indicated, with control vector being added to keep the total amount (300 ng) of transfected DNA constant in each condition. 48 h later, cells were challenged with VSV-Luc (MOI = 0.1) for 6 h (except the mock group) followed by cell lysis and luciferase assay. **(B)** Immunoblot analysis of transfected EAP30, EAP20 (using anti-HA), CBP, IRF3 (using anti-FLAG) and endogenous actin under experimental conditions of (A) for each transfection groups. **(C-E)** qPCR analysis of IFN-β (C), OASL (D), and IFN-λ1 (E) mRNA levels in HEK293 cells transfected with different plasmid combinations similar to panel (A) for 48 h. “*”, “**”, and “***” denote statistical differences exist as compared with empty vector-transfected cells with a *P*-value of < 0.05, < 0.01, and <0.001, respectively.

Activation of the IFN-β promoter occurs after activated IRF3 translocates to nucleus and associates with the CBP/p300 co-transcriptional factors. This transcriptional complex further binds to IFN-β promoter to drive up type I IFN transcription [38]. Since our earlier data showed that ectopic expression of EAP30 alone was insufficient in conferring antiviral activity (Fig 5C and 5D), we speculated that coordinate action of EAP30, IRF3 and CBP is required for activating antiviral responses. This hypothesis is mostly plausible for the functional significance of the interactions of EAP30 with IRF3 and CBP (Fig 7). To test this, we set up co-expression experiments in HEK293 cells while keeping the transfected plasmid DNA amount constant, followed by challenging cells with VSV-Luc to evaluate the antiviral protection (Fig 8A). The results showed that overexpression of EAP30, CBP, or IRF3 alone was not sufficient to confer antiviral activity as none inhibited replication of VSV-Luc compared to vector control. Co-expression of CBP and IRF3 reduced VSV-Luc replication by 50%, while co-expression of EAP30 with IRF3 or CBP induced negligible antiviral effect. Interestingly, when EAP30 was expressed together with CBP and IRF3, VSV-Luc replication was curtailed by ~75%. In contrast, we did not observe a synergistic effect when we replaced the EAP30 vector with an EAP20 construct in the triple transfection group. Immunoblotting data confirmed the successful expression of CBP, IRF3, EAP30, and EAP20 in each transfection conditions (Fig 8B). The antiviral effects were accompanied with expression of IRF3-dependent antiviral genes, IFN-β (Fig 8C), OASL (Fig 8D), and IFN-λ1 (Fig 8E), and the extent of antiviral gene upregulation was correlated with the observed antiviral effects. Specifically, substantial upregulation of the three representative IRF3 target genes was seen in cells co-transfected with IRF3 and CBP. Adding EAP30, but not EAP20, to the transfection cocktail enhanced the antiviral gene expression further (Fig 8C–8E), resulting in the most potent antiviral effect (Fig 8A, the last column from the left). Collectively, these data demonstrate that EAP30 synergizes with IRF3 and CBP to activate the IFN antiviral response, suggesting that EAP30 is required for optimal induction of IRF3-dependent innate antiviral defense.

## Discussion

In this study, we have uncovered a novel role for the ESCRT-II subunit EAP30 in IRF3-dependent innate immune responses to viral infections. We found that depletion of EAP30 dampens the induction of type I and type III IFNs, ISGs and chemokines via TLR3 and RLR signaling pathways, impairing the establishment of an antiviral state. Importantly, this previously unappreciated effect of EAP30 was observed in multiple cell types, including non-neoplastic hepatocytes PH5CH8, hepatoma Huh7-TLR3 and Huh7.5-TLR3 cells, and HEK293 cells. It also held true in distinct viral infection settings, i.e., following infection by SeV, HCV or VSV, or stimulation by poly(I:C), a viral dsRNA surrogate. Interestingly, unlike its conventional role in the ESCRT pathway and its emerging role in viral budding, EAP30 was found to operate in the nucleus rather than cytoplasm to fulfill its function in facilitating IRF3-mediated antiviral defense. Specifically, our data demonstrate that EAP30 forms a complex with IRF3 and its transcriptional co-activator, CBP (Fig 7), and these interactions are pivotal for controlling transcription of antiviral genes, as depletion of EAP30 resulted in profound reduction in virus-induced IRF3 binding to CBP (Fig 6E) and to target gene promoters such as IFN-β, IFN-λ1 and ISG56 (Fig 6F). Lending further support to this notion, overexpression of EAP30 together with IRF3 and CBP activated expression of antiviral genes and protected cells against VSV challenge (Fig 8). Taken together, our data suggest that EAP30 is an essential factor that bridges the collaborative action of IRF3 and CBP in the nucleus in inducing innate antiviral responses.

Several lines of evidence suggest that EAP30 acts independent of the ESCRT-II complex to facilitate IRF3-mediated innate immune responses. First, knockdown of EAP20, another component of the ESCRT-II complex, had no demonstrable effect on induction of IRF3 target genes (Figs 1 and 2). Second, EAP20 was not associated with IRF3 or CBP, regardless of viral infection status (Fig 7). Third, overexpression of EAP20 failed to act in synergy with ectopically co-expressed IRF3 and CBP to drive up IFN antiviral responses, as opposed to the effect of EAP30 (Fig 8). Fourth, while EAP30 knockdown inhibited activation of the IFN-β promoter downstream of both TLR3 and RLR pathways (Fig 2), depletion of the third ESCRT-II component, EAP45, undermined the RLR pathway (Fig 2B) but left TLR3 signaling intact (Fig 2A). Consistent with this, our data showed that EAP45 acts downstream of RIG-I, MDA5, and MAVS but had no effect on IFN activation by the IRF3 kinases, TBK1 and IKKε, or by IRF3-5D, a constitutively active IRF3 mutant mimicking phosphorylated IRF3 (Fig 2). Preliminary experiments indicated that EAP45 is required for activation of NF-κB downstream of the RLR-MAVS pathway (S17 Fig). Further studies are needed to understand the precise role of EAP45 in RLR signaling.

The activation of IRF3, characterized by its C-terminal phosphorylation, dimerization and subsequent nuclear translocation, has been extensively studied. A plethora of information is available concerning the upstream PRRs, adaptors, kinases and other regulators that control IRF3 activation. In contrast, relatively little is known about the molecular mechanisms by which activated IRF3 operates in the nucleus to induce transcription of IFNs and ISGs of direct IRF3 target. Our data have clearly shown that EAP30 regulates a step after IRF3 activation, as knockdown of EAP30 had no appreciable effect on virus-induced phosphorylation (Fig 6A and 6D), dimerization (Fig 6B), or nuclear translocation (Fig 6C) of IRF3, while inhibiting IFN induction by IRF3-5D (Fig 2A). The lack of an effect on activation of NF-κB-dependent promoter (Fig 3A) and upregulation of NF-κB-dependent and IRF3-independent genes (Fig 3C and S8 Fig) is also consistent with the fact that bifurcation of the two signaling branches occurs prior to the kinases that activate these transcription factors. The data that a fraction of EAP30 localizes in the nucleus (Fig 6C) and is required for virus-induced association of IRF3 and CBP (Fig 6E) and IRF3 binding to its target promoters (Fig 6F) strongly argue nucleus as the location where EAP30 exerts its effect on IRF3-mediated innate immune responses. Of note, a role for ESCRT-II proteins in regulating nuclear processes is not unexpected. It has been reported that ESCRT-II plays role in gene transcription in the nucleus by associating with the elongation factor for RNA Polymerase II (ELL) and increasing the catalytic rate of transcription elongation by Pol II [39–41]. It should be noted, however, the novel role of EAP30 in IRF3-dependent innate immune responses revealed in our study is by no means a non-specific effect on gene transcription. As noted above, EAP30 depletion did not impact viral induction of genes solely controlled by NF-κB (Fig 3C and S8 Fig). Knockdown of EAP30 had no demonstrable effect on ISG expression and establishment of an antiviral state induced by IFN-α through the Jak-Stat signaling, either (Fig 4E and S12 Fig).

What is the exact role of EAP30 in regulating IRF3-mediated innate immune responses? We found overexpression of EAP30 *per se* did not augment the antiviral phenotypes (Figs 5C and 5D and 8), which may be explained by the data that the endogenous EAP30 was already abundantly expressed. Alternatively, additional factors/processes may be involved. Our data demonstrate EAP30 forms a complex with IRF3 and CBP in the nucleus (Fig 7) and is required for virus-induced association of IRF3 and CBP and IRF3 binding to target promoters (Fig 6E and 6F), suggesting a model in which EAP30 may bridge the IRF3-CBP interactions, or perhaps helps maintain certain conformations of IRF3 and/or CBP, thereby facilitating the formation of so-called “enhanceosome” that assembles on and activates IFN-β and IFN-λ promoters. Supporting this hypothesis, we found that EAP30 bound both IRF3 and CBP in nuclear extracts and the associations were notably stronger after SeV infection (Fig 7). In addition, when EAP30 was ectopically co-expressed with IRF3 and CBP, IFN antiviral responses ensued and VSV replication was inhibited (Fig 8), supporting the notion that EAP30 acts in synergy with IRF3 and CBP. It should be noted that CBP is known to bind to a variety of transcriptional factors including the STATs and NF-κB [42]. Further studies will be needed to determine why EAP30 was required for IRF3-dependent gene transcription but dispensable for expression of genes downstream of NF-κB or Jak-STAT signaling.

At present, we do not know the precise mechanism that confers EAP30 the ability to synergize the action of IRF3 and CBP on activating the IFN response. Possible scenarios include that EAP30, IRF3, and CBP may assemble in different manners and possibly recruit additional factors to the promoter after virus infection. Clearly, overexpression of EAP30 alone was not sufficient to activate IFN production (Fig 8), and the distribution of EAP30 in the nucleus vs cytoplasm did not exhibit an obvious change before and after SeV infection (Fig 6C, S13 and S16 Figs). It will be interesting to determine in future studies whether EAP30 undergoes post-translational modifications and/or conformational changes after viral infection that alters its ability to interact with IRF3 and CBP.

In summary, this is the first study that demonstrates the ESCRT-II subunit EAP30 is involved in IRF3-dependent innate immune responses by facilitating the IRF3 binding to CBP and its target gene promoters. Our data provide novel insights into nuclear processes that regulate IRF3-mediated antiviral gene expression. Conceivably, exploiting or enhancing EAP30-mediated signaling may lead to new antiviral strategies for combating infectious diseases. Conversely, targeting EAP30 for inhibition may open new avenues of treating autoimmune diseases associated with aberrant IFN responses.

## Materials and methods

### Cells, viruses, and reagents

PH5CH8 non-neoplastic hepatocytes (provided by Nobuyuki Kato, Okayama University, Japan) [43], human hepatoma Huh7.5-TLR3 and Huh7-TLR3 cells that were stably reconstituted for the expression of human TLR3 (developed in this laboratory) [29, 30], and human embryo kidney HEK293 cells (obtained from American Type Culture Collection), were maintained as described previously [28, 29, 44]. HCV (JFH1 strain) was produced by transfecting Huh7.5 cells with *in vitro*-transcribed JFH1 RNA and infectious HCV titers were determined as described [29, 45]. VSV-GFP was a gift from Sean Whelan (Harvard University). Sendai virus (SeV, Cantell strain) was obtained from Charles River Laboratories. Poly(I:C) was purchased from Sigma (Sigma-Alrich, St. Louis, MO). For stimulation of cells, poly(I:C) was added directly into culture medium at a final concentration of 10–20 μg/ml to engage the TLR3 pathway or complexed with Lipofectamine 2000 (Invitrogen, Carlsbad, CA) (at 1:2 ratio) before adding to the cells to elicit RLR signaling. For HCV and SeV infections, cells were infected with JFH1 virus (MOI = 0.1) and with 160 hemagglutinin units (HAU)/ml of SeV, respectively. A human CCL5/RANTES DuoSet ELISA kit (R & D systems) was used to measure RANTES production in cell culture supernatants. Production of biologically active human IFNs in cell culture supernatants was determined by a microplaque reduction assay using VSV on Vero cells, as described previously [46].

### Plasmids

Plasmid vectors encoding human TRIF, RIG-I, and MAVS have been described previously [28, 44, 47, 48]. The following plasmids were provided by their indicated contributors: pcDNA3-FlagTBK1 and pcDNA3-FlagIKKε (Kate Fitzgerald) [4]; pEFBos-RIG-I-N (encoding the N-terminal 229 amino acids of RIG-I) and pEFBos-FlagMDA5 (Takashi Fujita) [9]; pGFP-IRF3-5D (Rongtuan Lin) [13]. pcDNA3β-FLAG-CBP-HA (Addgene, Cambridge, MA, plasmid #32908) [49]. The reporter plasmids pIFN-β-Luc [13], PRDII-Luc [50], (PRDIII-I)_4_-Luc [51], and pIRF3(-779)-Luc containing human IRF3 promoter upstream of *firefly* luciferase reporter gene [31] were kind gifts from Rongtuan Lin, Michael Gale, Christina Ehrhardt, and Paula Pitha-Rowe, respectively. pRL-TK (Promega, Madison, WI) was used to normalize transfection efficiencies. Plasmid DNAs were transfected into cells using Lipofectamine 2000 as per the manufacturer's instructions.

To generate expression constructs for human EAP20 and EAP30, full-length cDNA encoding EAP20 or EAP30 was amplified from PH5CH8 cells by RT-PCR with the following gene-specific primers: EAP20-EcoRI-ClaI-F (5’- ctctaGAATTCATCGATATGGCGATGAGTTTCGAGTGG -3’) and EAP20-KpnI-NheI-R (5’- taaGCTAGCGGTACCCTAGAAGAACTTGACGCCTC-3’) for the EAP20 construct; EAP30-EcoRI-F (5’-ttGAATTCATGCACCGCCGCGGGGTGGGAG-3’) and EAP30-KpnI-R (5’-aaGGTACCTCAGGGGAGGGCTTCTCTGGC-3’) for the EAP30 plasmid. The cDNA fragment of the EAP20 or EAP30 gene was cloned between the *EcoRI* and *Kpn I* restriction sites of the pCAGGS-HA mammalian expression vector (a gift from Shaobo Xiao). The target gene sequences in the pCAGGS-HA-EAP30 and pCAGGS-HA-EAP20 recombinant plasmids were confirmed by DNA sequencing.

### RNA interference

To knock down the expression of individual ESCRT-II subunits, siRNA duplexes targeting human EAP20, EAP30, and EAP45 were synthesized by GE Dharmacon (Fisher Scientific, Pittsburgh, PA) with sequences as follows: siEAP30, CUUGCAGAGGCCAAGUAUA(UU) [22]; siEAP20, GUCGAUCCAGAUUGUAUUA(UU) and siEAP45, GGAAUAUUGCAGGUGCCUU(UU) [25]. Cells (2–4 x10^5^ cells per well in 6-well plates) were transfected with 200 μM siRNA by Lipofectamine 2000 (Invitrogen) for 48 h followed by indicated stimuli. In each experiment, the efficiency of siRNA silencing was evaluated by qPCR. To stably knock down EAP30 in HEK293 cells, pLKO.1-shSNF8#27 carrying a human EAP30 shRNA with the target sequence of ATCTTGACTGACATCCTGGGC (GE Dharmacon, TRCN0000015727) was used for lentivirus packaging and transduction of cells. Following selection in medium containing 2 μg/ml of puromycin, survived cells were pooled and their phenotypes were characterized by immunoblotting.

### Quantitative PCR (qPCR)

Total cellular RNA was extracted using TRIzol (Invitrogen) and subsequently used for synthesis of cDNA. The abundance of RANTES, IP-10, IFN-β, IFN-λ1, IFN-λ2/3, OASL, Mx1, PKR, ISG15, IL-6, IL-8, MIP-1β and 28S (or β-actin, internal controls) transcripts, and HCV RNA levels were analyzed by qPCR using gene-specific primers (S1 Table) and GoTaq qPCR Master Mix (Promega) on an iCycler IQ5 real-time PCR system (Bio-Rad, Hercules, CA). For PCR detection, after enzyme activation of 2 minutes at 95°C, 40 cycles of 15 seconds at 95°C and 1 minute at 60°C were performed. Cq values were obtained by using the formula 2-ΔCq. The relative abundance of each target gene was determined by normalization to endogenous 28S or β-actin mRNA. Fold change in expression of each target gene in response to the indicated stimulus was calculated by comparing with mock-stimulated cells.

### Luciferase reporter assay

The promoter activities of IFN-β, PRDII, PRDIII-I, and IRF3(-779) were determined by cotransfecting cells with pIFN-β-Luc, pPRDII-Luc, pPRDIII-I-Luc, or pIRF3(-779)-Luc, respectively, together with the pRL-TK plasmid that expresses *Renilla* luciferase as an internal control, as described previously [29, 47]. Briefly, cells (4 x10^4^ cells per well in 48-well plates) were cotransfected with the indicated promoter-reporter plasmid (80 ng) and pRL-TK (20 ng), with/without addition of 100–200 ng of an expression vector encoding a gene of interest. Twenty-four hours later, cells were mock-treated or infected with SeV for 8 h, then lysed and assayed for *firefly* luciferase and *Renilla* luciferase activities. Data are expressed as mean relative luciferase activity plus standard deviation for one representative experiment carried out in triplicate of at least three independent experiments.

### Immunoblotting (IB)

Immediately before harvesting, cells were washed with cold phosphate-buffered saline (PBS). Whole cell lysates were prepared using a modified radioimmunoprecipitation assay (RIPA) buffer (50 mM Tris-HCl, pH 7.4, 150 mM NaCl, 1% NP-40, 0.1% SDS, 5 mM EDTA, 0.5% sodium deoxycholate) supplemented with protease inhibitor cocktails (Sigma-Alrich). Whole cell extracts were quantified and subjected to electrophoresis and immunoblot analysis as previous described [28, 48]. The following monoclonal (mAb) or polyclonal (pAb) primary antibodies were used: mouse anti-FLAG M2 mAb at 1:1,000 (Stratagene, La Jolla, CA); anti-actin mAb at 1:10,000 (Sigma-Alrich); anti-ISG15 mAb at 1:1000, anti-IFIT3 mAb at 1:2000, anti-TRAF1 mAb at 1:1000, anti-p100/p52 mAb at 1:1000, anti-IRF3 mAb at 1:2,000, anti-CBP mAb at 1:200, and anti-EAP30 mAb at 1:1,000 (Santa Cruz Biotechnology, Dallas, TX); and anti-HA (12CA5, hybridoma supernatant) at 1:100; rabbit anti-IRF3 pAb at 1:10,000 (a gift from Michael David, University of California-San Diego); rabbit anti-phospho-Ser396-IRF3 at 1:1,000 (Cell signaling Technology, Danvers, MA); rabbit anti-CBP pAb at 1:200 (Santa Cruz); rabbit anti-ISG56 pAb at 1:500 [30]; goat anti-MDA5 pAb at 1:200 (Abcam); rabbit anti-SeV pAb at 1:10,000 (a gift from Ilkka Julkunen, National Public Health Institute, Helsinki); rabbit anti-SNF8 (EAP30) pAb at 1:300 (Proteintech, Chicago, IL). The secondary antibodies were peroxidase-conjugated goat anti-rabbit or anti-mouse IgGs (Southern Biotech, Birmingham, AL). Protein bands were visualized using Immobilon Western Chemiluminescent HRP substrate (Milipore, Billerica, MA), followed by exposure to X-ray film.

### Immunoprecipitation (IP)

Cells (2–4 x10^6^) cultured in 10-cm dish were transfected and stimulated as indicated. At time of harvesting, cells were lysed in a buffer containing 50 mM HEPES (pH 7.4), 1.5 mM EDTA, 150 mM NaCl, 10% glycerol, 10 mM NaF, 1 mM Na_3_VO_4_, 0.5 mM dithiothreitol (DTT), 1% Triton X-100, and protease inhibitor cocktails, and clarified by centrifugation. Six hundred micrograms of each sample were subjected to IP. Samples were pre-cleared with protein A/G PLUS agarose (Santa Cruz) and control IgG for 1–2 hours at 4°C and then supernatant were incubated for overnight with anti-CBP antibodies (1:50) or rabbit anti-IRF3 pAb (1:500) at 4°C before incubation with protein A/G PLUS agarose for additional 4 hours. Protein-antibody immune complexes were washed with PBS containing 0.5% NP-40 and dissolved in SDS loading buffer and subsequently subjected to immunoblot analysis as described above.

### Indirect immunofluorescence staining

Cells (4 x10^4^) grown in 8-well chamber slides were transfected with indicated siRNA for 48 h prior to mock infection or infection with SeV for additional 16 h. Cells were fixed with 3.7% formaldehyde, permeabilized in 0.2% Triton X-100, and subsequently immunostained with rabbit anti-IRF3 pAb at 1:400 or mouse anti-EAP30 mAb (1:100) followed by Alexa Fluor 488-conjugated anti-rabbit secondary antibody (Invitrogen) or Texas Red-conjugated anti-mouse secondary antibody (Southern Biotech) at 1:200. After counterstaining the nuclei with 4', 6-diamidino-2-phenylindole (DAPI), slides were mounted and examined by fluorescence microscopy.

### Native PAGE for IRF3 dimerization assay

Twenty micrograms of each protein sample were fractionated on 7.5% native acrylamide gel as described previously [52]. After electrophoresis, proteins were transferred to nitrocellulose membrane and analyzed by immunoblotting. IRF3 dimer and monomer were detected by rabbit anti-IRF3 pAb.

### Cytoplasmic and nuclear protein extraction

Cytoplasmic and nuclear extracts were prepared from cells following indicated stimulus. Briefly, cells grown in 10-cm dish were washed with cold PBS and scraped off in 1 ml of PBS. The cells were pelleted at 3,000 rpm for 30 seconds at 4°C. To extract cytoplasmic proteins, a low salt buffer was added (50 mM HEPES pH 7.9, 10 mM KCl, 1 mM EDTA pH 8.0, 1 mM EGTA pH 8.0, 1 mM Na_3_VO_4_, 1mM Na_4_P_2_O_7_, 20 mM NaF, 1 mM DTT, 0.5 mM PMSF, and protease inhibitor cocktails) and cells were left on ice for 10 min before adding 1% NP-40 to the suspension. After centrifugation at 6,000 rpm for 30 seconds, the supernatant was collected and kept as cytoplasmic extract (CE). To further extract nuclear proteins, pellets were washed in 1.0 M sucrose in low salt buffer and centrifuged at 12,000 rpm for 10 min at 4°C. The pellets were then lysed in a high salt buffer (50 mM HEPES pH 7.9, 400 mM KCl, 1 mM EDTA pH 8.0, 1 mM EGTA pH 8.0, 20 mM NaF, 1 mM Na_3_VO_3_, 1mM Na_2_P_2_O_7_, 10% glycerol, 1 mM DTT, 0.5 mM PMSF, and protease inhibitor cocktails) and vortexed at 4°C for 20 min. The nuclear extract (NE) was collected from supernatant after 10 min of centrifugation.

### Chromatin immunoprecipitation (ChIP)

HEK293-shEAP30 and HEK293-shCon cells (1.5x10^7^ cells cultured in 15-cm dish) mock-infected or infected with SeV were cross-linked with 1% formaldehyde at room temperature for 10 min. The reaction was then stopped by adding 0.125 M glycine. ChIP assays were performed using the ChIP-ITTM Express kit (Active Motif, Carlsbad, CA) according to the manufacturer’s instructions. In brief, the cells were harvested and suspended in a hypotonic lysis buffer (10 mM Tris-HCl, pH 7.5, 10 mM KCl, 2mM MgCl2, 2.5 mM sodium pyrophosphate, 1 mM beta-glycerolphosphate, 0.5% NP-40, protease inhibitor cocktails) to prepare the nuclei. After centrifugation, nuclei were resuspended in a nuclear lysis buffer (50 mM Tris-HCl, pH 8.1, 10 mM EDTA, 1% SDS, protease inhibitor cocktails) and lysates were sonicated to generate DNA fragments with an average length of ~300 bp. After removal of cell debris by centrifugation, supernatants were pre-cleared with magnetic beads and control IgG for 1 h at 4°C. Following removal of the beads, lysates were incubated for overnight with 2 μg of rabbit IgG control, rabbit anti-IRF3 pAb, or rabbit anti-p65 plus the beads. The immune complexes were collected, washed four times, and subsequently eluted in elution buffer containing 1% SDS and 0.05 M NaHCO3. Crosslinks were then broken in 250 mM NaCl by incubation at 65°C for 2.5 h. The resulting samples were treated with 10 mM EDTA, 40mM Tris-HCl, pH 6.5 and 200 μg/ml of proteinase K at 37°C for 1 h and DNA was purified with the MiniElute PCR purification kit (Qiagen, Hilden, Germany). The immunoprecipitated DNAs and input DNA were analyzed by qPCR for IRF3-binding motifs in IFNB, IFNL1, and IFIT1 promoters, or for NF-κB-binding motifs in IL8, CXCL1, and IL32 promoters, using GoTaq Q-PCR mixture (Promega) as described above. The sense and antisense primers for amplifying the putative IRF3-binding and NF-κB-binding sequences in each promoter are listed in S2 Table.

### Statistical analysis

Data analysis was performed using GraphPad Prism v6.0g software (Graphpad). Statistical differences were determined using a Two-tailed Student *t* test. Data are expressed as mean ± standard deviations (SD) of data from at least three sample replicates. Differences with a *P*-value of < 0.05 are considered statistically significant.

## Supporting information

S1 Fig Knockdown of ESCRT-II subunits by siRNAs. (A) qPCR analysis of EAP20, EAP30, and EAP45 mRNAs in PH5CH8 cells transfected with control siRNA (siCon) or siRNA targeting EAP20, EAP30 or EAP45 for 48 h, followed by mock-stimulation or stimulation by poly(I:C) for 6 h or SeV for 8 h. Data are expressed as mean ± standard deviations (SD) from three sample replicates. “*” and “**” denote statistical differences exist as compared with negative control siRNA-transfected cells with a *P*-value of < 0.05 and < 0.01, respectively. (B) Immuoblotting of HA-EAP20, HA-EAP30, or HA-EAP45 expression by anti-HA antibody in PH5CH8 cells co-transfected with the indicated siRNA and HA-tagged EAP construct for 48 h.
(PDF)

S2 Fig Knockdown of EAP30 significantly impairs virus-induced RANTES production. PH5CH8 cells were transfected with control siRNA (siCon) or EAP30 siRNA. Forty-eight hours later, cells were mock-infected or infected with SeV for 8 h. Production of RANTES in culture supernatants was quantified by ELISA. “**” denotes statistical difference exists as compared with negative control siRNA-transfected cells with a *P*-value of < 0.01.
(PDF)

S3 Fig Intracellular Sendai virus (SeV) mRNA levels in cells transfected with siRNAs targeting individual ESCRT-II subunits. PH5CH8 cells were transfected with EAP20, EAP30, EAP45, or a non-targeting control siRNA. Forty-eight hours later, cells were mock-infected (empty bars) or infected with SeV for 8 h (solid bars). The relative abundance of SeV, EAP20, EAP30, and EAP45 mRNAs was determined by qPCR (normalized to 28S rRNA level). “*” and “**” denote statistical differences exist as compared with negative control siRNA-transfected cells with a *P*-value of < 0.05 and <0.01, respectively.
(PDF)

S4 Fig Knockdown of ESCRT-II subunits by siRNAs in Huh7.5-TLR3 cells. Cells were transfected with control siRNA or siRNA targeting EAP20, EAP30 or EAP45 for 48 h, followed by mock-treatment or stimulation by poly(I:C) for 6 h. For the HCV infection groups, cells were infected with HCV for 8 h prior to siRNA transfection for additional 48 h. “*” and “**” denote statistical differences exist as compared with negative control siRNA-transfected cells with a *P*-value of < 0.05 and < 0.01, respectively.
(PDF)

S5 Fig Intracellular HCV RNA levels in HCV-infected Huh7.5-TLR3 cells transfected with siRNAs targeting individual ESCRT-II subunits. Cells were mock-infected (empty bars) or infected with HCV (filled bars) for 8 h, followed by transfection with the indicated EAP siRNA, or a negative control siRNA, for additional 48 h. The relative abundance (normalized to 28S) of intracellular HCV RNA, EAP20, EAP30, and EAP45 was determined by qPCR. “*” and “**” denote statistical differences exist as compared with negative control siRNA-transfected cells with a *P*-value of < 0.05 and < 0.01, respectively.
(PDF)

S6 Fig Immunoblot analysis of ectopically expressed signaling proteins upstream of IFN-β induction in PH5CH8 cells transfected with negative control siRNA or indicated EAP siRNA under conditions of experiments shown in Fig 2. (PDF)

S7 Fig Knockdown of EAP30 does not affect endogenous IRF3 mRNA expression. PH5CH8 cells were transfected with control siRNA, IRF3 siRNA (as a positive control), or EAP30 siRNA for 48 h, followed by mock-stimulation (empty bars), or stimulation by poly(I:C) for 6 h (grey bars), or infected with SeV for 8 h (black bars). The expression levels of IRF3 and EAP30 mRNAs were quantitated by qPCR. “*” and “**” denote statistical differences exist as compared with negative control siRNA-transfected cells with a *P*-value of < 0.05 and < 0.01, respectively.
(PDF)

S8 Fig Knockdown of EAP30 does not affect virus-induced expression of genes of strict NF-κB target. PH5CH8 cells were transfected with control siRNA (siCon) or EAP30 siRNA. Forty-eight hours later, cells were mock-infected or infected with SeV for 8 h. The mRNA levels of indicated NF-κB target genes (a total of 7) and EAP30 were quantified by qPCR (normalized to β-actin).
(PDF)

S9 Fig Knockdown of EAP30 significantly curtails virus-induced ISG expression. PH5CH8 cells were transfected with control siRNA (siCon) or EAP30 siRNA. Forty-eight hours later, cells were mock-infected or infected with SeV for 8 h. The expression levels of indicated ISGs were quantified by qPCR (normalized to β-actin). “*” and “**” denote statistical differences exist as compared with negative control siRNA-transfected cells with a *P*-value of < 0.05 and < 0.01, respectively.
(PDF)

S10 Fig Knockdown of EAP30 impairs virus-induced expression of ISG proteins but not proteins of strict NF-κB target. Pools of HEK293 cells that were stably transduced with a scrambled non-targeting shRNA (shCon) or EAP30 shRNA (shEAP30) were mock-infected or infected with SeV for 16 h. The expression levels of three ISGs (ISG15, IFIT3 and MDA5), two well-characterized NF-κB targets (P100 and TRAF1), EAP30 and actin were examined by immunoblotting.
(PDF)

S11 Fig Proliferation rates and viability of HEK293 cells stably expressing control (293-shCon) or EAP30 (293-shEAP30) shRNA. 293-shCon and 293-shEAP30 cells were plated into 60-mm dishes (200,000 cells per dish). The cells were examined for cell number and viability (by trypan blue exclusion) for six consecutive days. Each data point represented mean ± standard deviations (SD) of data from triplicate wells.
(PDF)

S12 Fig Knockdown of EAP30 does not alter induction of ISG proteins by IFN, nor does it affect the establishment of an antiviral state by IFN. (A) Immunoblot analysis of ISG15, IFIT3 and actin expression in 293-shCon and 293-shEAP30 cells that were mock-stimulated or stimulated by indicated concentrations of IFN-α. (B) 293-shCon and 293-shEAP30 cells were mock-stimulated or stimulated by 250 U/ml IFN-α. Sixteen hours later, cells were mock-infected or infected with VSV-Luc (MOI = 0.3) for 6 h. Viral replication levels in lysed cells were quantified by firefly luciferase assay.
(PDF)

S13 Fig Endogenous EAP30 localizes to both cytoplasm and nucleus as examined by confocal fluoresence microscopy. PH5CH8 cells were mock-infected (upper panels) or infected with SeV (lower panels) for 16 h. Cells were fixed and immunostained with mouse anti-EAP30 (red fluorescence). Nuclei were counterstained blue with DAPI.
(PDF)

S14 Fig Virus-induced IRF3 nuclear accumulation is not altered by EAP30 knockdown. PH5CH8 cells transfected with control (left) or EAP30 (right) siRNA for 48 h were infected with SeV for additional 16 h. Cells were fixed and immunostained with rabbit anti-IRF3 (green fluorescence). The percentage of cells with predominant nuclear IRF3 was shown at the lower right corner in each panel.
(PDF)

S15 Fig ChIP assay of NF-κB (p65) binding to IL8, CXCL1 and IL32 promoters in 293-shCon and 293-shEAP30 cells that were mock-infected or infected by SeV. (PDF)

S16 Fig EAP30 localizes to both cytoplasmic and nuclear fractions, regardless of viral infection status. Cytoplasmic (CE) and nuclear extracts (NE) were prepared from HEK293FT cells that were transfected with HA-EAP30 plasmid or control vector for 48 h and subsequently mock-infected or infected with SeV for additional 16 h. Immunoblot analysis of EAP30 (using anti-HA), IRF3, SeV, lamin A/C (nuclear protein marker), β-tubulin (cytoplasmic protein marker), and actin (loading control) is shown.
(PDF)

S17 Fig Knockdown of EAP45 compromises viral induction of NF-κB-dependent genes. (A) SeV-induced PRDII promoter activities in PH5CH8 cells transfected with control siRNA or siRNA targeting EAP45 or EAP20. (B) qPCR analysis of IL-6 and IL-8 mRNA levels in PH5CH8 cells transfected with the indicated siRNA and mock-infected or infected with SeV. “*” denotes *P*<0.05 as compared with control siRNA-transfected cells.
(PDF)

S1 Table qPCR primers for gene expression analysis. (DOC)

S2 Table qPCR primers for ChIP assay. (DOC)
