# Supplementary figures and images for: Pivotal role for the ESCRT-II complex subunit EAP30/SNF8 in IRF3-dependent innate antiviral defense
Source: PLoS Pathog. 2017 Oct 30;13(10):e1006713. doi: 10.1371/journal.ppat.1006713 (PMC5679654; doi:10.1371/journal.ppat.1006713)

**A**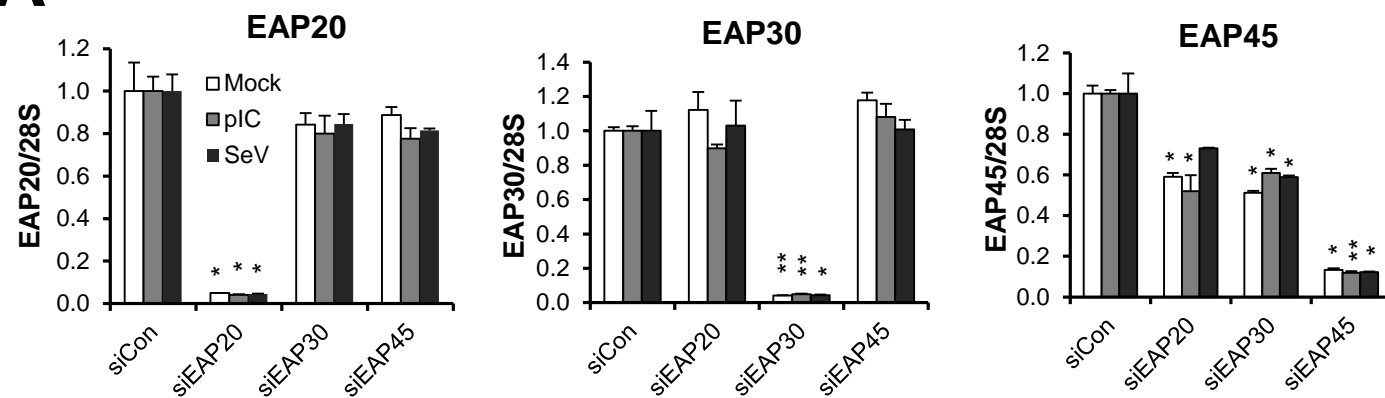**B**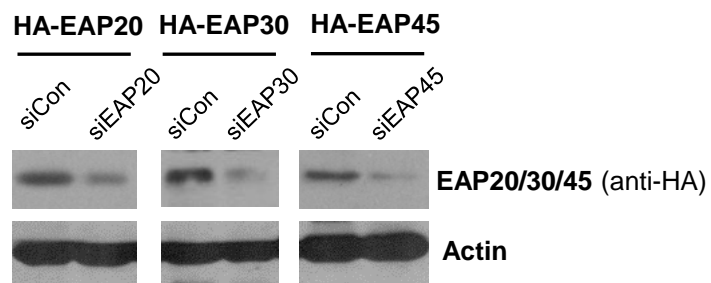

Supplement: S1 Fig — (A) qPCR analysis of EAP20, EAP30, and EAP45 mRNAs in PH5CH8 cells transfected with control siRNA (siCon) or siRNA targeting EAP20, EAP30 or EAP45 for 48 h, followed by mock-stimulation or stimulation by poly(I:C) for 6 h or SeV for 8 h. Data are expressed as mean ± standard deviations (SD) from three sample replicates. “*” and “**” denote statistical differences exist as compared with negative control siRNA-transfected cells with a P-value of < 0.05 and < 0.01, respectively. (B) Immuoblotting of HA-EAP20, HA-EAP30, or HA-EAP45 expression by anti-HA antibody in PH5CH8 cells co-transfected with the indicated siRNA and HA-tagged EAP construct for 48 h. (PDF) [file ppat.1006713.s001.pdf]

## RANTES

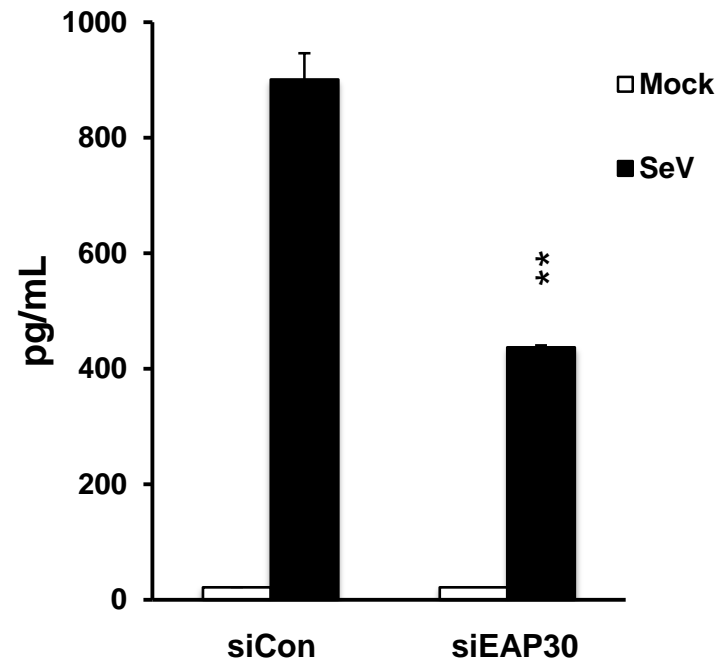

Supplement: S2 Fig — PH5CH8 cells were transfected with control siRNA (siCon) or EAP30 siRNA. Forty-eight hours later, cells were mock-infected or infected with SeV for 8 h. Production of RANTES in culture supernatants was quantified by ELISA. “**” denotes statistical difference exists as compared with negative control siRNA-transfected cells with a P-value of < 0.01. (PDF) [file ppat.1006713.s002.pdf]

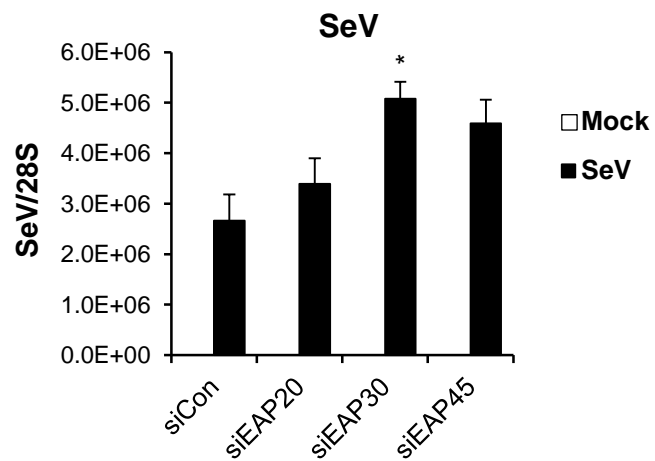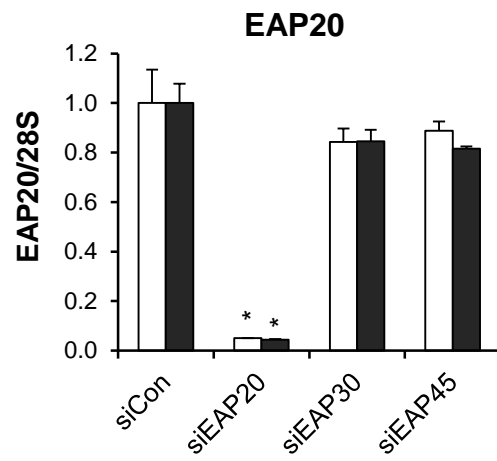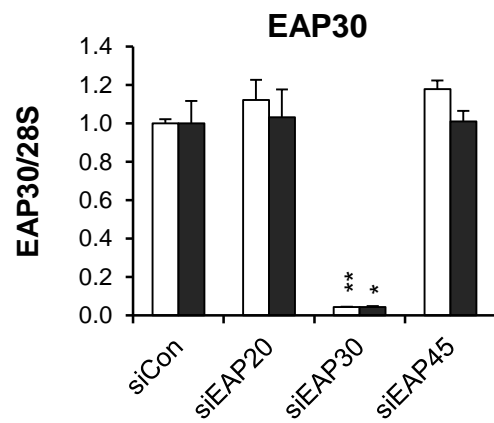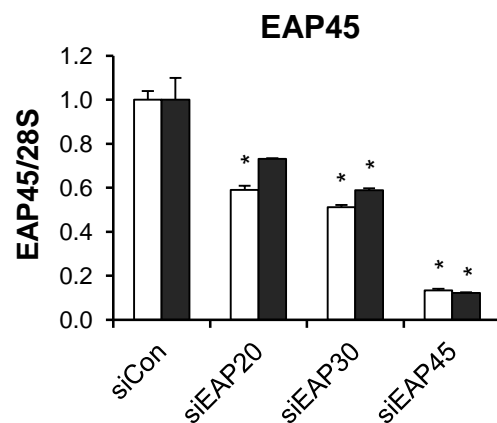

Supplement: S3 Fig — PH5CH8 cells were transfected with EAP20, EAP30, EAP45, or a non-targeting control siRNA. Forty-eight hours later, cells were mock-infected (empty bars) or infected with SeV for 8 h (solid bars). The relative abundance of SeV, EAP20, EAP30, and EAP45 mRNAs was determined by qPCR (normalized to 28S rRNA level). “*” and “**” denote statistical differences exist as compared with negative control siRNA-transfected cells with a P-value of < 0.05 and <0.01, respectively. (PDF) [file ppat.1006713.s003.pdf]

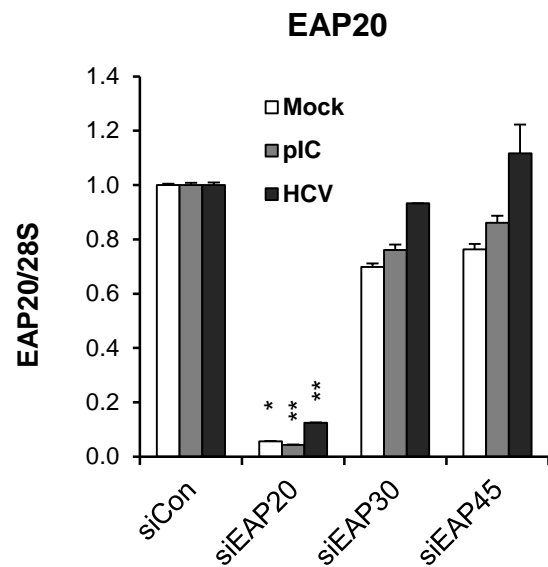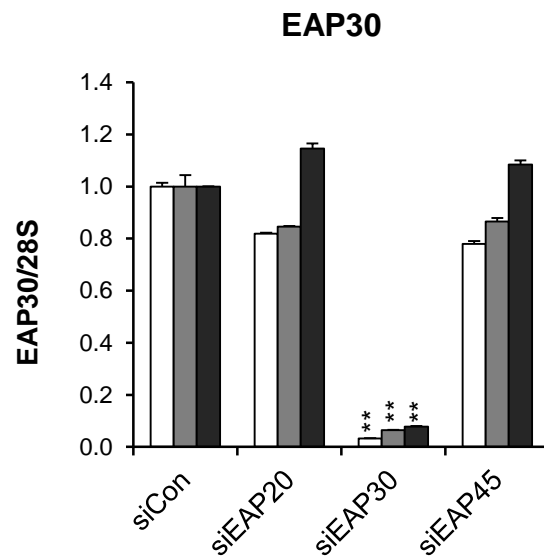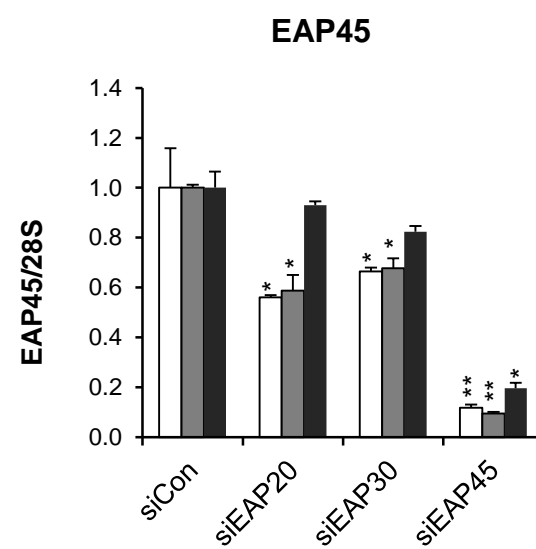

Supplement: S4 Fig — Cells were transfected with control siRNA or siRNA targeting EAP20, EAP30 or EAP45 for 48 h, followed by mock-treatment or stimulation by poly(I:C) for 6 h. For the HCV infection groups, cells were infected with HCV for 8 h prior to siRNA transfection for additional 48 h. “*” and “**” denote statistical differences exist as compared with negative control siRNA-transfected cells with a P-value of < 0.05 and < 0.01, respectively. (PDF) [file ppat.1006713.s004.pdf]

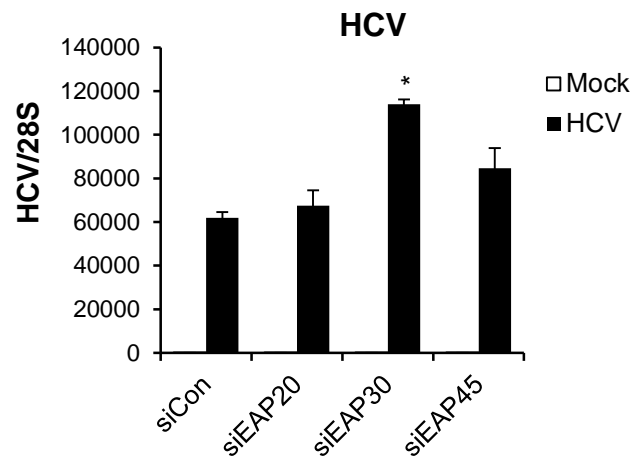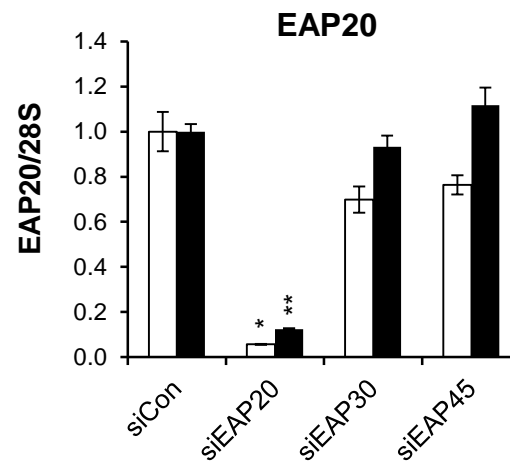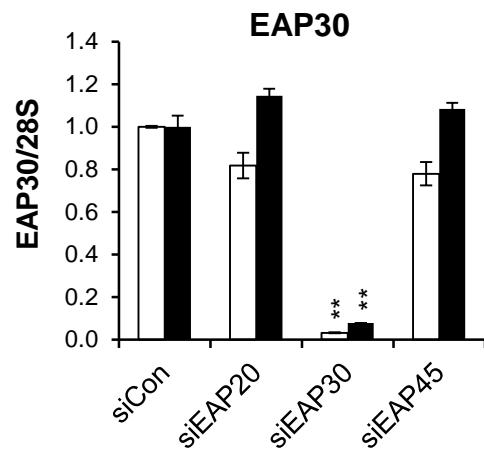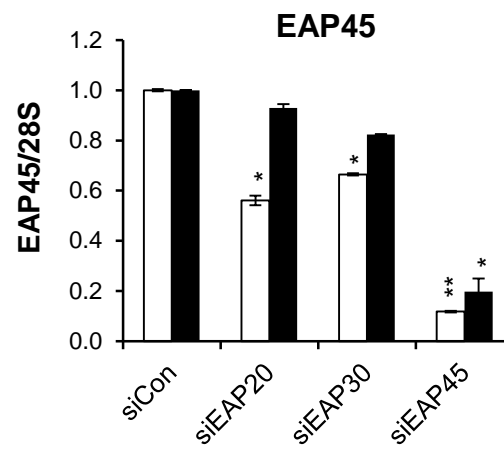

Supplement: S5 Fig — Cells were mock-infected (empty bars) or infected with HCV (filled bars) for 8 h, followed by transfection with the indicated EAP siRNA, or a negative control siRNA, for additional 48 h. The relative abundance (normalized to 28S) of intracellular HCV RNA, EAP20, EAP30, and EAP45 was determined by qPCR. “*” and “**” denote statistical differences exist as compared with negative control siRNA-transfected cells with a P-value of < 0.05 and < 0.01, respectively. (PDF) [file ppat.1006713.s005.pdf]

**A**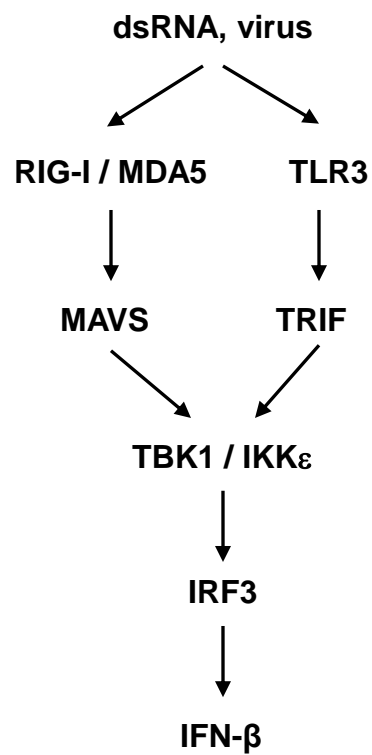**B**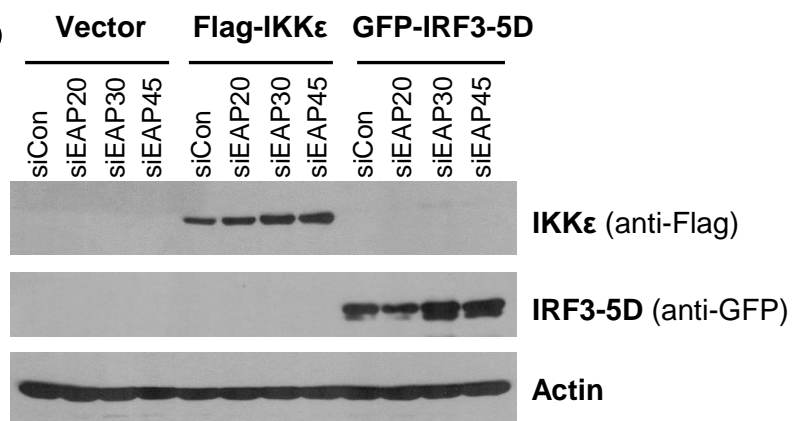**C**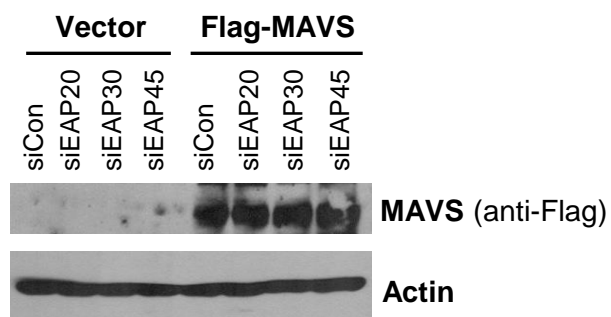

Supplement: S6 Fig — (PDF) [file ppat.1006713.s006.pdf]

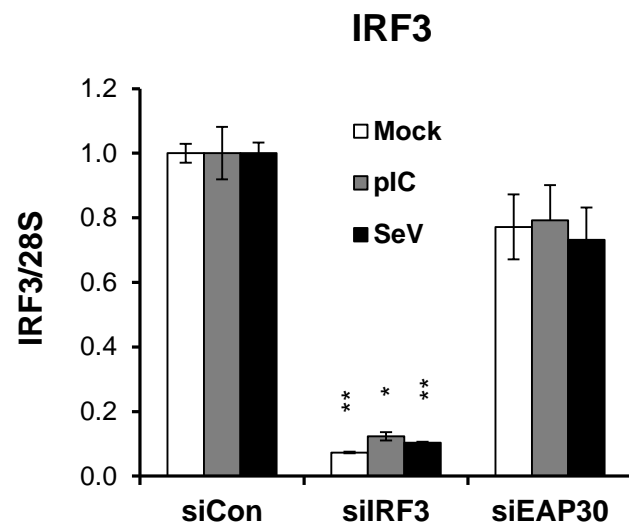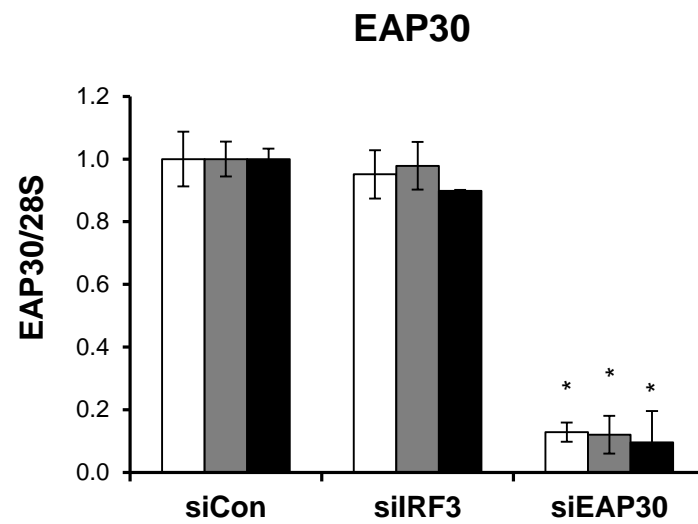

Supplement: S7 Fig — PH5CH8 cells were transfected with control siRNA, IRF3 siRNA (as a positive control), or EAP30 siRNA for 48 h, followed by mock-stimulation (empty bars), or stimulation by poly(I:C) for 6 h (grey bars), or infected with SeV for 8 h (black bars). The expression levels of IRF3 and EAP30 mRNAs were quantitated by qPCR. “*” and “**” denote statistical differences exist as compared with negative control siRNA-transfected cells with a P-value of < 0.05 and < 0.01, respectively. (PDF) [file ppat.1006713.s007.pdf]

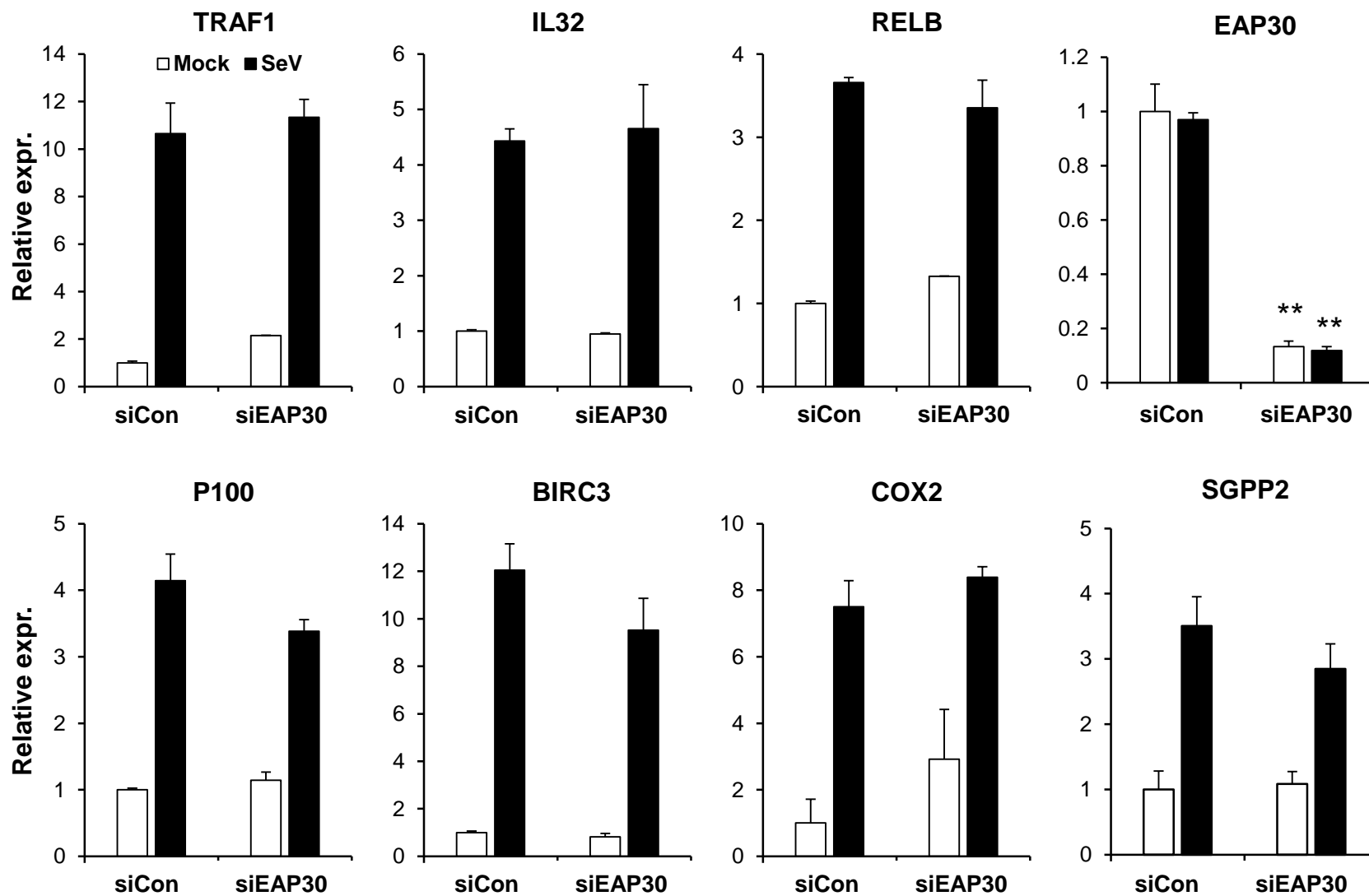

Supplement: S8 Fig — PH5CH8 cells were transfected with control siRNA (siCon) or EAP30 siRNA. Forty-eight hours later, cells were mock-infected or infected with SeV for 8 h. The mRNA levels of indicated NF-κB target genes (a total of 7) and EAP30 were quantified by qPCR (normalized to β-actin). (PDF) [file ppat.1006713.s008.pdf]

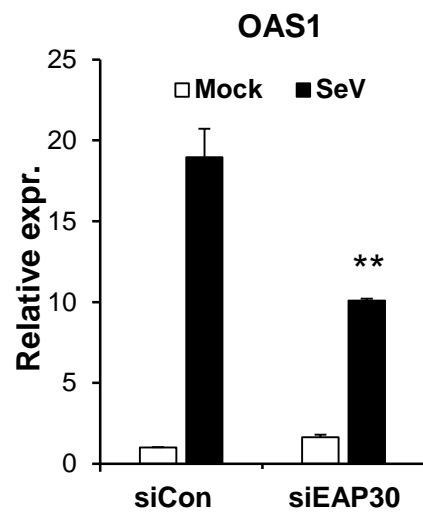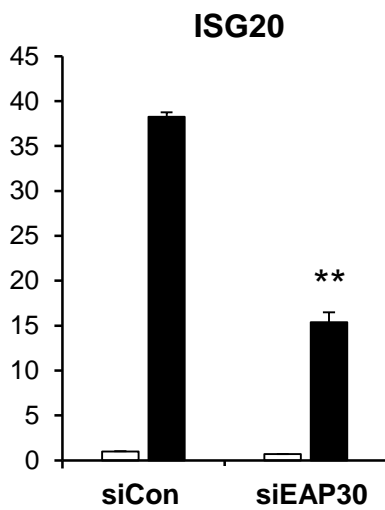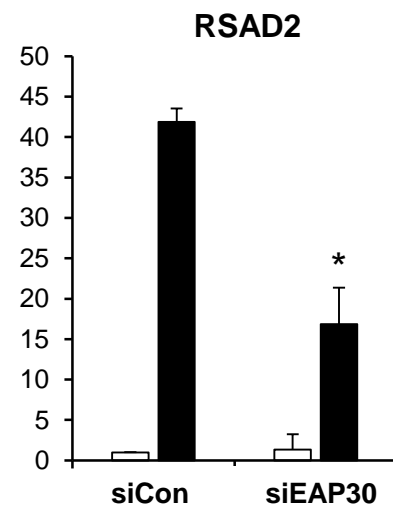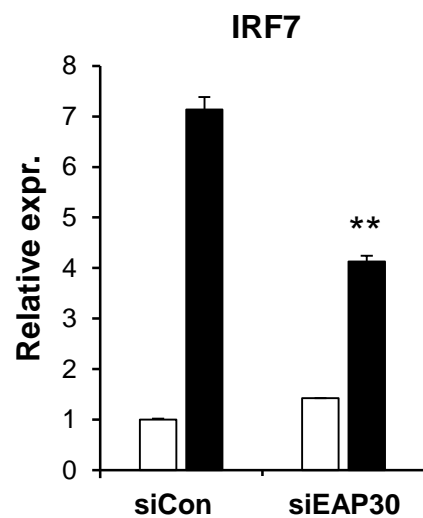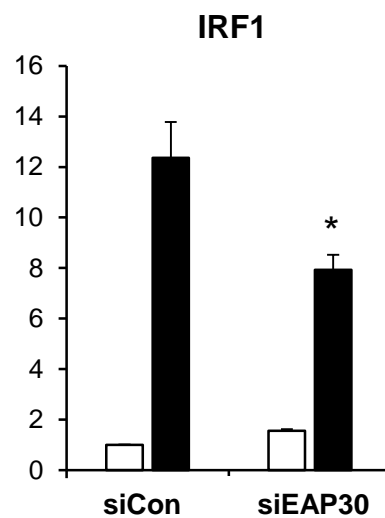

Supplement: S9 Fig — PH5CH8 cells were transfected with control siRNA (siCon) or EAP30 siRNA. Forty-eight hours later, cells were mock-infected or infected with SeV for 8 h. The expression levels of indicated ISGs were quantified by qPCR (normalized to β-actin). “*” and “**” denote statistical differences exist as compared with negative control siRNA-transfected cells with a P-value of < 0.05 and < 0.01, respectively. (PDF) [file ppat.1006713.s009.pdf]

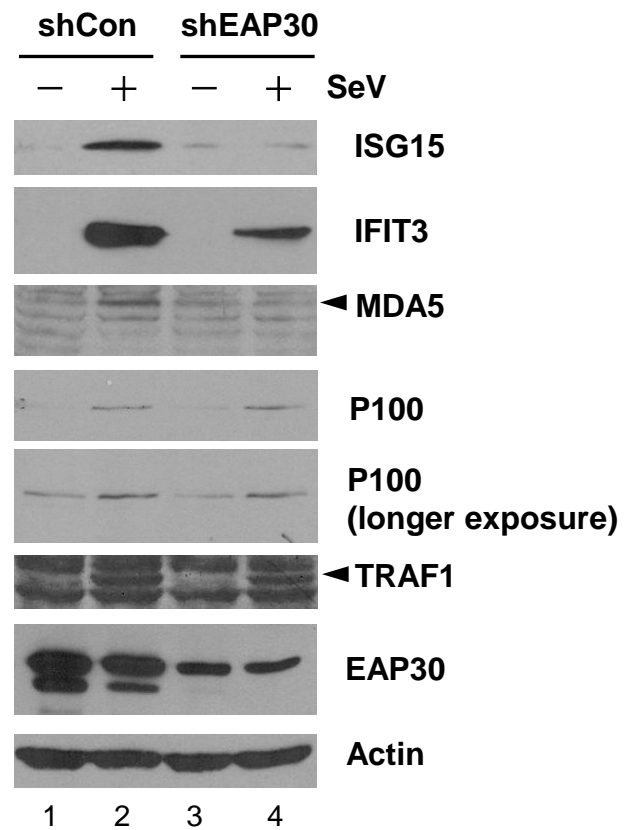

Supplement: S10 Fig — Pools of HEK293 cells that were stably transduced with a scrambled non-targeting shRNA (shCon) or EAP30 shRNA (shEAP30) were mock-infected or infected with SeV for 16 h. The expression levels of three ISGs (ISG15, IFIT3 and MDA5), two well-characterized NF-κB targets (P100 and TRAF1), EAP30 and actin were examined by immunoblotting. (PDF) [file ppat.1006713.s010.pdf]

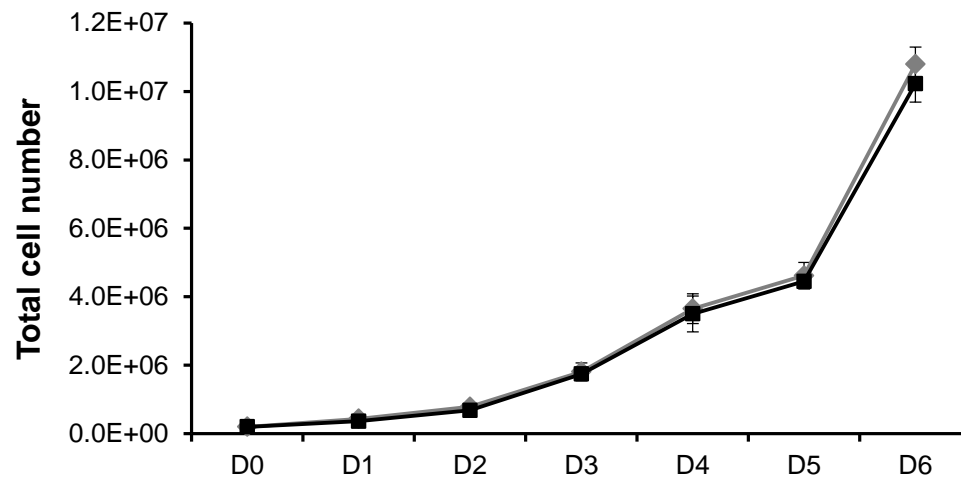

**Cell confluency (%)** 10 15 20 30-35 60-65 75-80 90-95

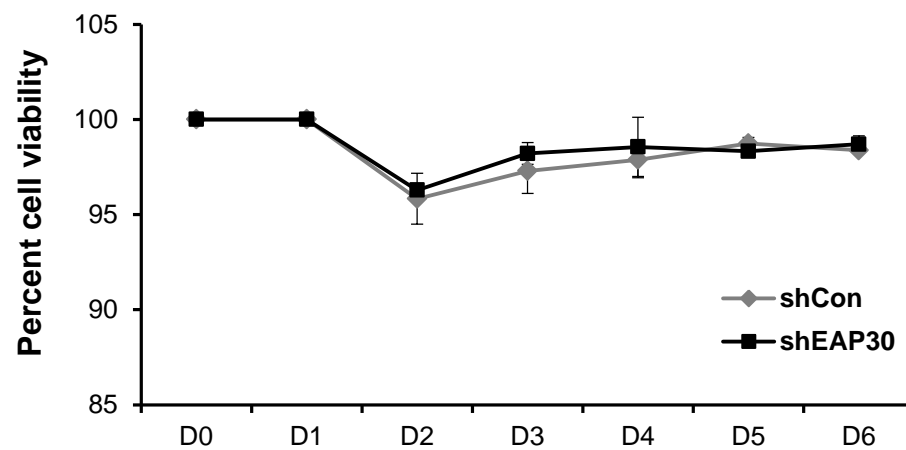

Supplement: S11 Fig — 293-shCon and 293-shEAP30 cells were plated into 60-mm dishes (200,000 cells per dish). The cells were examined for cell number and viability (by trypan blue exclusion) for six consecutive days. Each data point represented mean ± standard deviations (SD) of data from triplicate wells. (PDF) [file ppat.1006713.s011.pdf]

**A**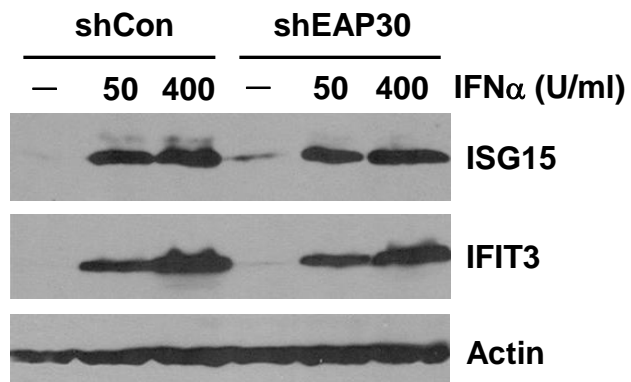**B**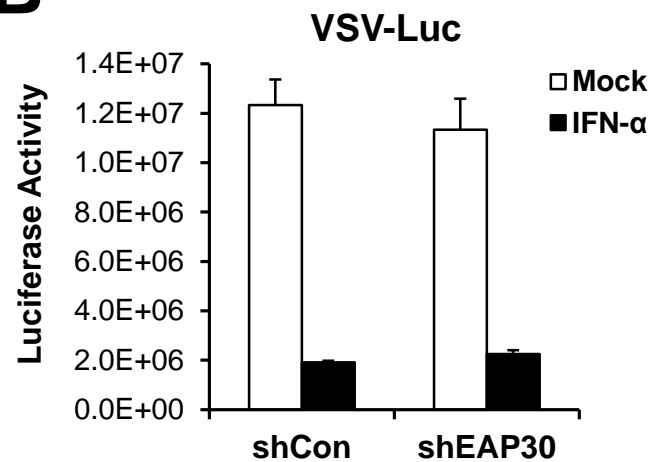

Supplement: S12 Fig — (A) Immunoblot analysis of ISG15, IFIT3 and actin expression in 293-shCon and 293-shEAP30 cells that were mock-stimulated or stimulated by indicated concentrations of IFN-α. (B) 293-shCon and 293-shEAP30 cells were mock-stimulated or stimulated by 250 U/ml IFN-α. Sixteen hours later, cells were mock-infected or infected with VSV-Luc (MOI = 0.3) for 6 h. Viral replication levels in lysed cells were quantified by firefly luciferase assay. (PDF) [file ppat.1006713.s012.pdf]

**Mock**

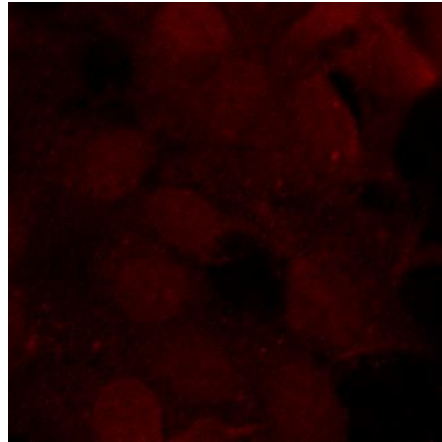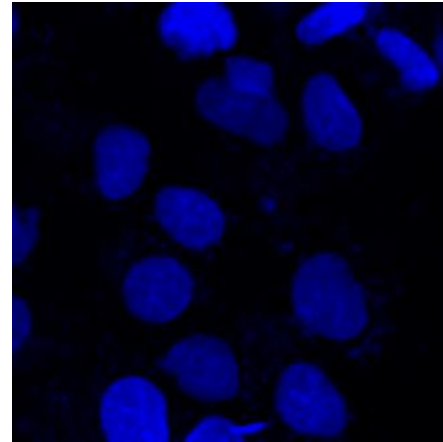

**SeV**

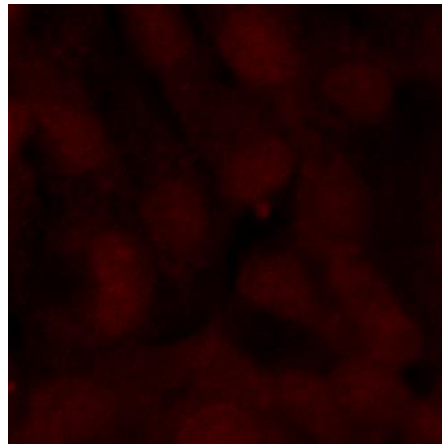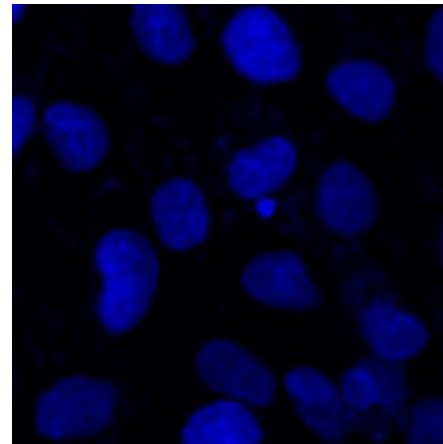

**EAP30**

**Nuclei**

Supplement: S13 Fig — PH5CH8 cells were mock-infected (upper panels) or infected with SeV (lower panels) for 16 h. Cells were fixed and immunostained with mouse anti-EAP30 (red fluorescence). Nuclei were counterstained blue with DAPI. (PDF) [file ppat.1006713.s013.pdf]

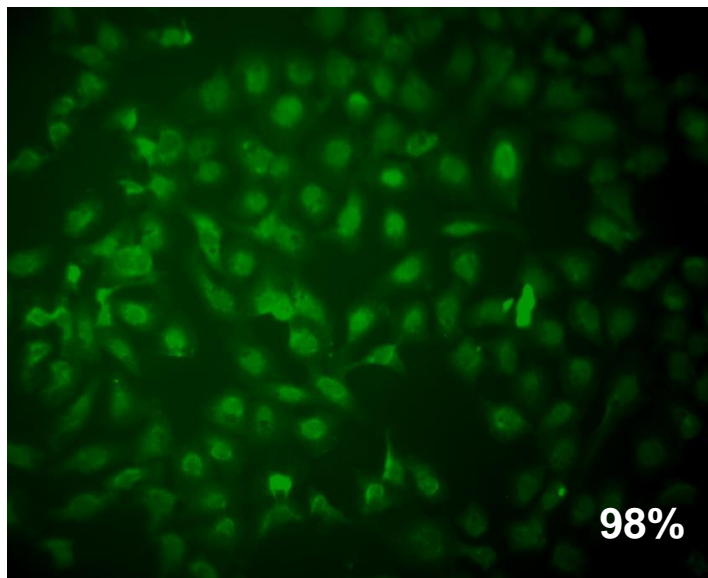

**siCon, SeV**

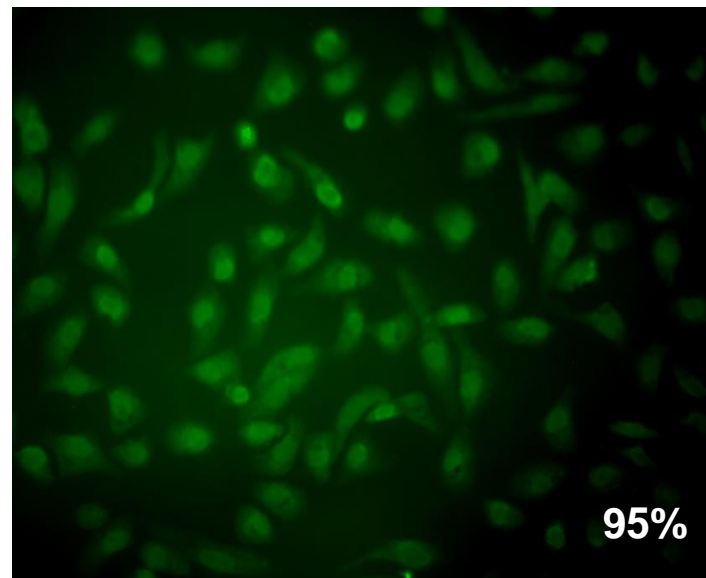

**siEAP30, SeV**

Supplement: S14 Fig — PH5CH8 cells transfected with control (left) or EAP30 (right) siRNA for 48 h were infected with SeV for additional 16 h. Cells were fixed and immunostained with rabbit anti-IRF3 (green fluorescence). The percentage of cells with predominant nuclear IRF3 was shown at the lower right corner in each panel. (PDF) [file ppat.1006713.s014.pdf]

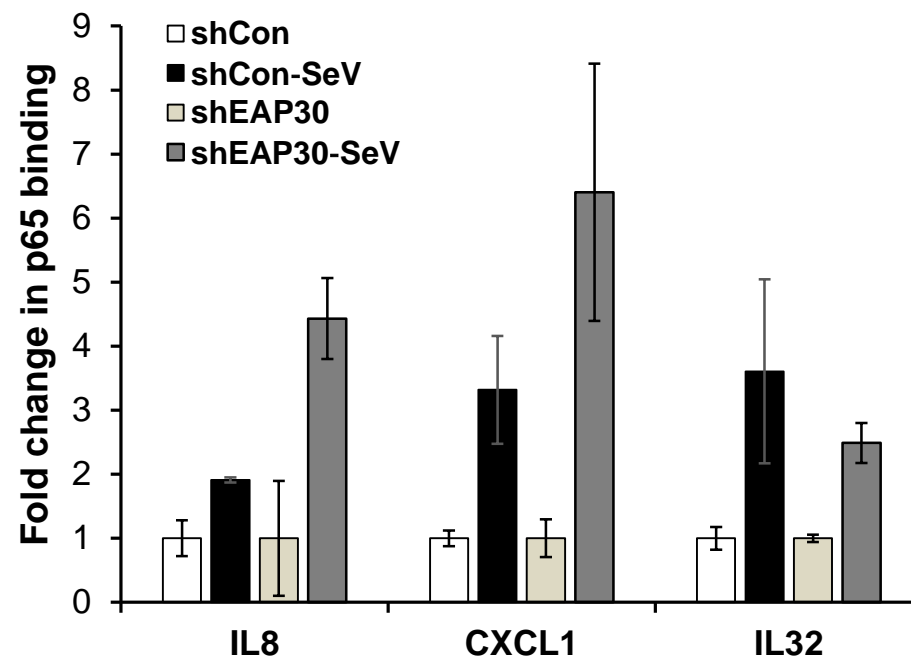

Supplement: S15 Fig — (PDF) [file ppat.1006713.s015.pdf]

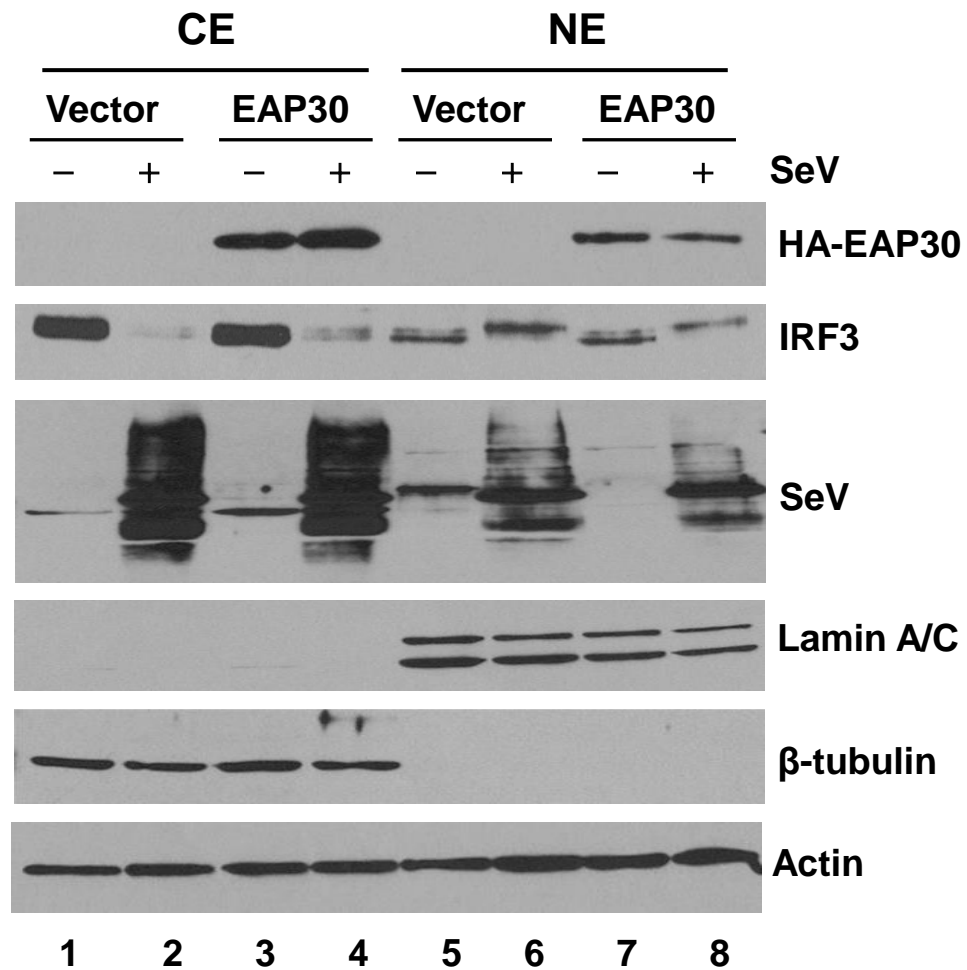

Supplement: S16 Fig — Cytoplasmic (CE) and nuclear extracts (NE) were prepared from HEK293FT cells that were transfected with HA-EAP30 plasmid or control vector for 48 h and subsequently mock-infected or infected with SeV for additional 16 h. Immunoblot analysis of EAP30 (using anti-HA), IRF3, SeV, lamin A/C (nuclear protein marker), β-tubulin (cytoplasmic protein marker), and actin (loading control) is shown. (PDF) [file ppat.1006713.s016.pdf]

**A**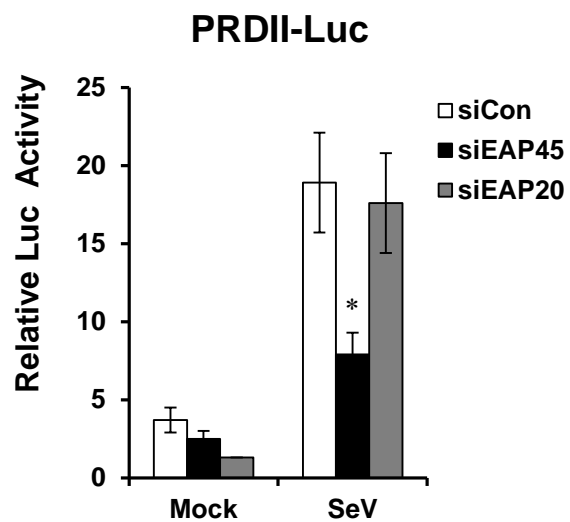**B**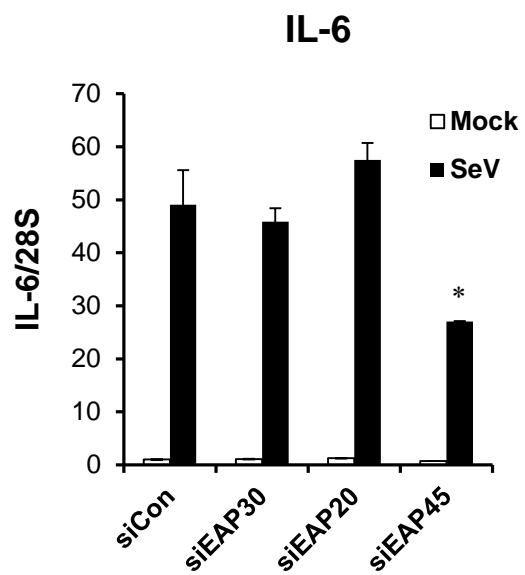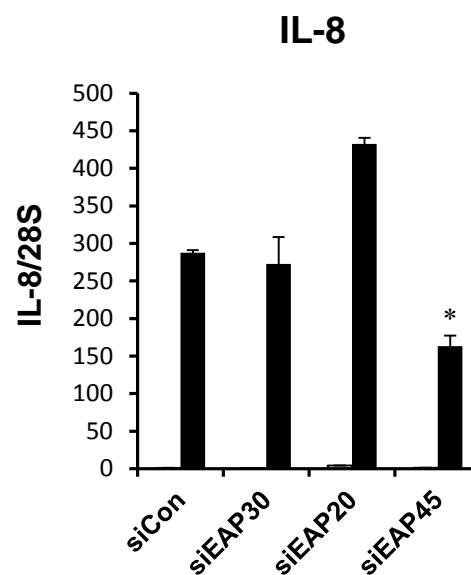

Supplement: S17 Fig — (A) SeV-induced PRDII promoter activities in PH5CH8 cells transfected with control siRNA or siRNA targeting EAP45 or EAP20. (B) qPCR analysis of IL-6 and IL-8 mRNA levels in PH5CH8 cells transfected with the indicated siRNA and mock-infected or infected with SeV. “*” denotes P<0.05 as compared with control siRNA-transfected cells. (PDF) [file ppat.1006713.s017.pdf]
