# Supplementary material for: Pivotal role for the ESCRT-II complex subunit EAP30/SNF8 in IRF3-dependent innate antiviral defense
Source: PLoS Pathog. 2017 Oct 30;13(10):e1006713. doi: 10.1371/journal.ppat.1006713 (PMC5679654; doi:10.1371/journal.ppat.1006713)
Supplement: S1 Table — (DOC) [file ppat.1006713.s018.doc]

**S1 Table. qPCR primers for gene expression analysis**

| **Target** | **Oligo name** | **Sequence (5′ - 3′)** |
| --- | --- | --- |
| 28S rRNA | 28S-F | gacccgctgaatttaagcat |
|  | 28S-R | gcctcgatcagaaggacttg |
| BIRC3 | BIRC3-F | tggtttccaaggtgtgagtacttg |
|  | BIRC3-R | gggctgtctgatgtggatagc |
| COX2 | COX2-F | tggcgctcagccatacag |
|  | COX2-R | tccgggtacaatcgcacttat |
| EAP20 | EAP20-F | ctgcacaaacagtccagcat |
|  | EAP20-R | tcgaggttccctttcttcct |
| EAP30 | EAP30-F | agaaacttgcagaggccaag |
|  | EAP30-R | attcttccggatctcctgct |
| EAP45 | EAP45-F | gccctggagacagtttcaga |
|  | EAP45-R | ctgagtcatcacggcaaaga |
| IFN-β | IFNβ-F | ccaacaagtgtctcctccaaatt |
|  | IFNβ-R | gtaggaatccaagcaagttgtagct |
| IFN-λ1 | IL29-F | gaagagtcactcaagctgaaaaactg |
|  | IL29-R | gagaagcctcaggtcccaatt |
| IFN-λ2/3 | IL28A/B-FQ-F | ggctctcccggatgcaa |
|  | IL28A/B-FQ-R | ggcatctttggccctcttaaa |
| IL6 | IL6-F | gctgcaggcacagaacca |
|  | IL6-R | actccttaaagctgcgcagaa |
| IL8 | IL8-QF | aagaaaccaccggaaggaac |
|  | IL8-QR | actccttggcaaaactgcac |
| IL32 | IL32-QF | cgacttcaaagagggctacc |
|  | IL32-QR | gagtgagctctgggtgctg |
| IP-10 | IP10-F | tgaaaaagaagggtgagaagagatg |
|  | IP10-R | cctttccttgctaactgctttcag |
| IRF1 | IRF1-FQ-F | ctgtcgccatgtgctgtca |
|  | IRF1-FQ-R | tgtccggcacaacttcca |
| IRF3 | IRF3-F | agaggctcgtgatggtcaag |
|  | IRF3-R | aggtccacagtattctccagg |
| IRF7 | IRF7-FQ-F | gccggctggaaaaccaactt |
|  | IRF7-FQ-R | tgagcgcgtacaccttgtgc |
| ISG15 | ISG15-QF | caccgtgttcatgaatctgc |
|  | ISG15-QR | ctttatttccggcccttgat |
| ISG20 | ISG20-409-F | ctcctgcacaagagcatcca |
|  | ISG20-474-R | cgttgccctcgcatcttc |
| MIP-1β | CCL4-F | cagcgctctcagcaccaa |
|  | CCL4-R | agcttcctcgcagtgtaagaaaa |
| MX1 | Mx1-F | caacctgtgcagccagtatga |
|  | Mx1-R | agcccgcagggagtcaat |
| OAS1 | OAS1-FQ-For | acctaacccccaaatctatgtcaa |
|  | OAS1-FQ-Rev | tggagaactcgccctctttc |
| OASL | OASL-F | tcttctcccacactcacatctatctg |
|  | OASL-R | caccatcaggattcttcacgaa |
| P100/P52 | P52-F | acgagggaccagccaagat |
|  | P52-R | tgcttgcccaccagactgt |
| PKR | PKR-FQ-F | tggttcttttgctactacgtgtgagt |
|  | PKR-FQ-R | ctgagaagtcaccttcagatgatga |
| RANTES | CCL5-F | ctgcatctgcctccccata |
|  | CCL5-R | gcgggcaatgtaggcaaa |
| RELB | RELB-F | gacccccatggcatcga |
|  | RELB-R | cgtggttgggcaggaagt |
| RSAD2 | Viperin-FQ-F | gagggccagatgagaccaaa |
|  | Viperin-FQ-R | gtgaagtgatagttgacgctggtt |
| SGPP2 | real-tSGPP2_ f | ggatgcatacggtcctggat |
|  | real-tSGPP2 _f | aacacaggaagaatggcacaact |
| TRAF1 | TRAF1-FQ-F | tctgtccaggctcgtcat |
|  | TRAF1-FQ-R | caacccccaatttgaagtccta |
| Sendai virus | Sendai-P-QF | ctctgggagaacaagcaagc |
|  | Sendai-P-QR | tcgcccagatcctgagatac |
| HCV (5’NTR) | HCV-FQ-F | ctcccctgtgaggaactactgtct |
|  | HCV-FQ-R | gaggctgtacgacactcatactaac |
