# Supplementary material for: Pivotal role for the ESCRT-II complex subunit EAP30/SNF8 in IRF3-dependent innate antiviral defense
Source: PLoS Pathog. 2017 Oct 30;13(10):e1006713. doi: 10.1371/journal.ppat.1006713 (PMC5679654; doi:10.1371/journal.ppat.1006713)
Supplement: S2 Table — (DOC) [file ppat.1006713.s019.doc]

**S2 Table. qPCR primers for ChIP assay**

| **Target promoter** | **Oligo name** | **Sequence (5′ - 3′)** |
| --- | --- | --- |
| IFN-β | IFNB_pro_F1 | tcgtttgctttcctttgctt |
|  | IFNB_pro_R1 | cagaggaatttcccactttca |
| IFN-λ1 | IFNL1_pro_F1 | ccaccacacctggctaattt |
|  | IFNL1_pro_R1 | ggagctgcatcaagaaggaa |
| IFIT1 (ISG56) | IFIT1_pro_F1 | agcaaccaaaaagcaaccag |
|  | IFIT1_pro_R1 | catcagtccattgctgccta |
| IL8 | IL8_pro_F1 | tgggccatcagttgcaaatc |
|  | IL8_pro_R1 | gtttgtgccttatggagtgctc |
| CXCL1 | CXCL1_pro_F1 | atctggaactccgggaatttcc |
|  | CXCL1_pro_R1 | atccgcgaaccccttttatg |
| IL32 | IL32_pro_F1 | ttggatcccacttggctgac |
|  | IL32_pro_R1 | agagggaaagtccagactcg |
